# Single-cell transcriptomics in MI identify Slc25a4 as a new modulator of mitochondrial malfunction and apoptosis-associated cardiomyocyte subcluster

**DOI:** 10.1038/s41598-024-59975-8

**Published:** 2024-04-23

**Authors:** Ting Zhou, Jing Pan, Kai Xu, Chenghui Yan, Jing Yuan, Haixu Song, Yaling Han

**Affiliations:** 1 Department of Cardiology, Union Hospital, Tongji Medical College, Huazhong University of Science and Technology, Wuhan, 430022 Hubei China; 2 State Key Laboratory of Frigid Zone Cardiovascular Disease, Cardiovascular Research Institute and Department of Cardiology, General Hospital of Northern Theater Command, Wenhua Road 83, Shenyang, 110016 Liaoning China; 3 School of Life Science and Biochemistry, Shenyang Pharmaceutical University, Shenyang, 110016 Liaoning China

**Keywords:** Single nuclei RNA sequencing, Mitochondrial malfunction, Apoptosis, Heart failure, Biological techniques, Cell biology, Computational biology and bioinformatics, Molecular biology, Biomarkers, Cardiology, Pathogenesis

## Abstract

Myocardial infarction (MI) is the leading cause of premature death. The death of cardiomyocytes (CMs) and the dysfunction of the remaining viable CMs are the main pathological factors contributing to heart failure (HF) following MI. This study aims to determine the transcriptional profile of CMs and investigate the heterogeneity among CMs under hypoxic conditions. Single-cell atlases of the heart in both the sham and MI groups were developed using single-cell data (GSE214611) downloaded from Gene Expression Omnibus (GEO) database (https://www.ncbi.nlm.nih.gov/geo/). The heterogeneity among CMs was explored through various analyses including enrichment, pseudo time, and intercellular communication analysis. The marker gene of C5 was identified using differential expression analysis (DEA). Real-time polymerase chain reaction (RT-PCR), bulk RNA-sequencing dataset analysis, western blotting, immunohistochemical and immunofluorescence staining, Mito-Tracker staining, TUNEL staining, and flow cytometry analysis were conducted to validate the impact of the marker gene on mitochondrial function and cell apoptosis of CMs under hypoxic conditions. We identified a cell subcluster named C5 that exhibited a close association with mitochondrial malfunction and cellular apoptosis characteristics, and identified Slc25a4 as a significant biomarker of C5. Furthermore, our findings indicated that the expression of Slc25a4 was increased in failing hearts, and the downregulation of Slc25a4 improved mitochondrial function and reduced cell apoptosis. Our study significantly identified a distinct subcluster of CMs that exhibited strong associations with ventricular remodeling following MI. Slc25a4 served as the hub gene for C5, highlighting its significant potential as a novel therapeutic target for MI.

## Introduction

Myocardial infarction (MI) is a life-threatening disease caused by coronary artery occlusion, leading to myocardial ischemia and ultimately resulting in cardiomyocyte (CM) death in the affected area^1^. MI complicated with heart failure (HF) is vulgar and is the leading cause of morbidity and mortality worldwide. The degree of MI is positively correlated with the risk of developing HF in patients^2^. Multiple therapeutic approaches based on inhibiting CMs death, controlling inflammation, and promoting heart regeneration have been proposed to protect MI patients from HF^3,4^. However, these therapeutic strategies cannot solve the root cause of CMs loss and reverse HF. Consequently, it is urgent to explore the pathogenesis of MI and intervene in ventricular remodeling after MI at an early stage.

Amounts of studies have revealed the mechanisms of MI induced HF at bulk RNA-sequencing level^1,5^. The traditional bulk RNA-sequencing only focuses on tissue resolution when studying MI, while single nuclei RNA sequencing (snRNA-seq) provides a new perspective for the study of MI by detecting the heterogeneity of various cells with single-cell resolution, identifying rare cell types, delineating cell subclusters, tracing cell lineages, locating mutated genes, and discovering new biomarkers. In recent years, increasing attention has been paid to investigate the mechanisms and therapeutic strategies of MI at single cell resolution. For example, Murilo et al. revealed that regulatory T cells with a specific affinity for myosin effectively suppress inflammation following MI, hinder neighboring T cells, and correlate with enhanced cardiovascular performance^6^. Kaiyu et al. demonstrated the heterogeneity and dynamics of immune cells during the course of MI and provided a theoretical foundation for the development of cell type specific interventions^7^. Jung et al. aimed to examine the temporal and spatial patterns of macrophage diversity and the possible involvement of Trem2^hi^ macrophages in the repair of cardiac tissue, offering significant findings regarding the immune response activated by MI^8^. The application of snRNA-seq technology in the heart focuses on immune cells, with limited research on parenchymal cells.

CMs are the most abundant cell type in the heart and individual CMs constitute the fundamental unit of gene regulation, understanding the heterogeneity and transcriptional program specific to each cell subcluster to determine its phenotype and function is crucial. Although, the cellular heterogeneity of CMs has been studied using snRNA-seq in several cardiac diseases. Li et al. showed that the heterogeneity of CMs in failing heart and non-failing heart was mainly reflected in myocardial contractility and metabolism^9^. Michail et al. revealed that CMs isolated from the hypertrophic heart exhibited heterogeneous transcriptional characteristic of hypoxic-induced responses^10^. However, there is still a lack of understanding regarding the gene programs that control ventricular remodeling and cardiac homeostasis. Consequently, it is important to uncover the mechanisms behind MI and HF, identify potential therapeutic targets, gain insights into the molecular underpinnings of dysfunctional CMs, and identify factors that regulate transitions between different cardiac states.

In our study, we constructed a CM atlas that included CMs from both sham and MI mice by integrating their snRNA-seq data. Through the analysis of pseudo time trajectories at different stages, the developmental trajectory of CMs was clearly characterized, and a CM subcluster that was common in the hearts of MI mice and closely related to mitochondrial malfunction as well as cell apoptosis was identified. On this basis, we identified a highly expressed gene *Slc24a5* that has not been reported in MI and further validated the role of Slc24a5 in promoting mitochondrial malfunction and cell apoptosis in CMs under hypoxic conditions. In conclusion, our study explores the mechanisms for MI induced cardiac remodeling, and provides a new potential treatment target for HF prevention.

## Materials and methods

### Public data sources

Public snRNA-seq dataset (GSE214611) and bulk RNA-seq dataset (GSE21610) were obtained from the Gene Expression Omnibus (GEO) database^11,12^.

### Quality control and data integration

Additional quality control measures were implemented on the cells by filtering for identified genes within the range of 300 to 5000 and mitochondrial gene percentage between 0 and 1%. The sample (GSM6613064) with low data quality (due to unusual cell proportion) was removed. Afterward, the integration of cells was performed utilizing the canonical correlation analysis (CCA) method, employing the 'IntegrateData()' function of the R package ‘Seurat’.

### Gene set functional analysis

To perform the gene set functional analysis, we utilized the R package ‘clusterProfiler’^13^ and ‘GSVA’^14^. Gene Set Variation Analysis (GSVA) and functional enrichment analysis were conducted using Gene Ontology (GO), Kyoto Encyclopedia of Genes and Genomes (KEGG)^15–17^, and the Reactome pathway databases. Hallmark gene sets and Reactome gene sets were obtained from R package ‘msigdbr’.

### Marker gene discovery

Differential expression analysis (DEA) was performed using the function findallmarkers() package in ‘Seurat’ to compare each cell subcluster with all others. We utilized the model (~ 0 + cluster + tissue) to contrast one cell subcluster versus all others, along with duplicate Correlation per individual sample, and further to obtain an estimate of the log2(fold change) between the given subcluster and all others. A threshold of log2(fold change) > 1 and* P* value < 0.05 was used. The dot plots showed a subset of significant genes.

### Pseudotime analysis

A single-cell pseudotime trajectory was constructed using the R package ‘slingshot’ (monocle3 v1.0.1)^18^. In order to perform dimension reduction, we utilized the UMAP technique, employing the ‘dimplot’ function to visualize the data. To identify differentially expressed genes, we employed the ‘graph_test’ function.

### Cell–cell communication analysis

Cell–cell communication analysis was performed based on the snRNA-seq data using R package ‘CellChat’ (CellChat v1.1.3)^19^. We randomly selected 500 cells from each subcluster of cells using the ‘subset’ function and utilized the Cellchat database, which incorporates information on ‘Secreted Signaling’, ‘ECM-Receptor’, and ‘Cell–Cell Contact’.

### Function score analysis

The mitochondrial dysfunction (MD) and apoptosis (AP) gene sets were obtained from the MSigDB signaling database. For detailed information, please refer to Supplementary File 1.

### Mice and MI surgery

The male C57BL/6 mice aged 10 weeks were obtained from Gempharmatech Co., Ltd.

Sham and left anterior descending branch (LAD) ligation surgery were performed as described in previously published protocols^20^. Temporarily anesthetize mice by inhaling 2% isoflurane. Make a small skin incision on the left chest, expose the heart, and permanently ligate the LAD with 7-0 silk thread. The animals undergoing sham surgery underwent the same procedure, but without ligating the LAD. All animal experiments were performed in accordance with the United Kingdom Animals (Scientific Procedures) Act 1986 and the American Veterinary Medical Association (AVMA) Guidelines for the Euthanasia of Animals (2020). Prior to the study, the research protocol was reviewed and approved by the Medical Ethics Committee of Union Hospital Affiliated to Huazhong University of Science and Technology. We affirmed that this study strictly followed the ARRIVE guidelines (https://arriveguidelines.org).

### Reagents

Antibodies used were as follows: Slc24a5 antibody (CST, #84835), Bcl2 antibody (Abcam #ab182858), Bax antibody (Abcam, #ab32503), PGC1α antibody (Abclonal, #A19674), MFN2 antibody (CST, #9482), β-actin antibody (Abcam, #ab8226), and GAPDH antibody (Abcam, #ab9485).

### Cell culture

CM lines (HL-1 and AC16) were seeded at a density of 1 × 10^6^ cells per well in six-well tissue culture plates and maintained in Dulbecco’s modified Eagle’s medium supplemented with 10% fetal bovine serum, 100 U/ml penicillin, and 100 U/ml streptomycin at 37 °C in a 5% CO_2_ atmosphere for 24 h. The cells were subjected to serum deprivation and incubated with Cocl2 (400 μM) for 24 h to mimic a hypoxic environment in the CMs. Subsequently, the CMs were transfected with Slc25a4 siRNA prior to treatment with Cocl2 for 24 h to inhibit the expression of Slc15a4 in hypoxic CMs.

### Western blotting

RIPA lysis buffer containing 1× protease inhibitor cocktail was employed to lyse the heart tissue and cultured cells at 0 °C for a minimum of 30 min. The protein concentration was assessed using the BCA Protein Assay Kit. The extracted protein, ranging from 20 to 40 µg, was electrophoresed on an SDS-PAGE gel and subsequently transferred onto a PVDF membrane. The membrane was subsequently blocked for 30 min with 5% skim milk. Next, the membrane was incubated overnight at 4 °C with specific primary antibodies. Afterward, peroxidase-conjugated secondary antibodies were added and incubated for 1 h at room temperature. Protein signals were detected using an Amersham Imager 680, and protein expression levels were quantified utilizing ImageJ software V1.8.0 (https://imagej.en.softonic.com/).

### RNA extraction and real-time polymerase chain reaction (RT-PCR)

The total RNA from left ventricular tissues was extracted using TRIzol reagent. Subsequently, reverse transcription into cDNA was achieved by utilizing the PrimeScript RT Kit with gDNA Eraser. Afterwards, the mRNA levels of the targeted genes were determined via quantitative PCR using SYBR1 Premix Ex Taq II and normalized to the housekeeping gene *GAPDH*, which served as an endogenous internal control. The primer sequences used in our study were as follows:

- *Slc25a4* (mice): Forward: 5′-CACTGTTCGTCGTAGGATGATGATG-3′; Reverse: 5′-GCAATCTTCCTCCAGCAGTCAAG-3′.
- *Slc25a4* (human): Forward: 5′-TGATTGTGTCGTGAGAATCCCC-3′;Reverse: 5′-AGAACTGCTTATGGCGATCCA-3′.
- *GAPDH* (mice): Forward: 5′-ACATCATCCCTGCATCCACT-3′; Reverse: 5′-GGGAGTTGCTGTTGAAGTCA-3′.
- *GAPDH* (human): Forward: 5′-TGGCCTTCCGTGTTCCTAC-3′; Reverse: 5′-GAGTTGCTGTTGAAGTCGCA-3′.

### TUNEL assay

The percentage of cell apoptosis was assessed using a TUNEL assay kit under the influence of Cocl2 stimulation, following the guidelines provided by the manufacturer.

### Flow cytometry

The cells were incubated with FITC Annexin V (BD Biosciences, #567356) and propidium iodide (PI) (BD Biosciences, #556463) for 30 min in the dark at room temperature. The apoptosis percentage of CMs was detected using a flow cytometer. The obtained data were analyzed using FlowJo software V1.8.0 (https://imagej.en.softonic.com/).

### Mito-tracker staining

To track the mitochondrial injury, Mito-Tracker staining (Thermo Fisher Scientific, #M22426 and #M7514) was employed. The samples were treated with a 200 nM concentration of Mito-Tracker for a duration of 15 min before photographing.

### Histological analysis

The hearts were cut into serial 5-μm-thick sections for wheat germ agglutinin (WGA) staining, immunohistochemistry, and immunofluorescence staining. The obtained data were analyzed using ImageJ software V1.8.0 (https://imagej.en.softonic.com/).

### Statistical analysis

Bioinformatics statistical analysis was performed using R language software v4.3.1. Molecular biology experimental data were presented as the mean ± standard error of the mean (SEM) of at least 3 independent experiments. Differences between the two groups were evaluated using the unpaired Student’s two-tailed *t*-test. Normal distribution of the data was analyzed using a Shapiro Wilk test or a Kolmogorov–Smirnov test. All data were analyzed using GraphPad Prism 8.3 (GraphPad Software, San Diego, CA). *P < 0.05; **P < 0.01; ns. indicates no significance between the 2 indicated groups.

### Ethics statements

All animal experiments were reviewed and approved by the Medical Ethics Committee of Union Hospital Affiliated to Huazhong University of Science and Technology. All animal experiments were performed in accordance with the United Kingdom Animals (Scientific Procedures) Act 1986 and the American Veterinary Medical Association (AVMA) Guidelines for the Euthanasia of Animals (2020). Prior to the study, the research protocol was reviewed and approved by the Medical Ethics Committee of Union Hospital Affiliated to Huazhong University of Science and Technology. We affirmed that this study strictly followed the ARRIVE guidelines (https://arriveguidelines.org).

## Results

### snRNA-seq data integration and clustering

**Figure 1:**
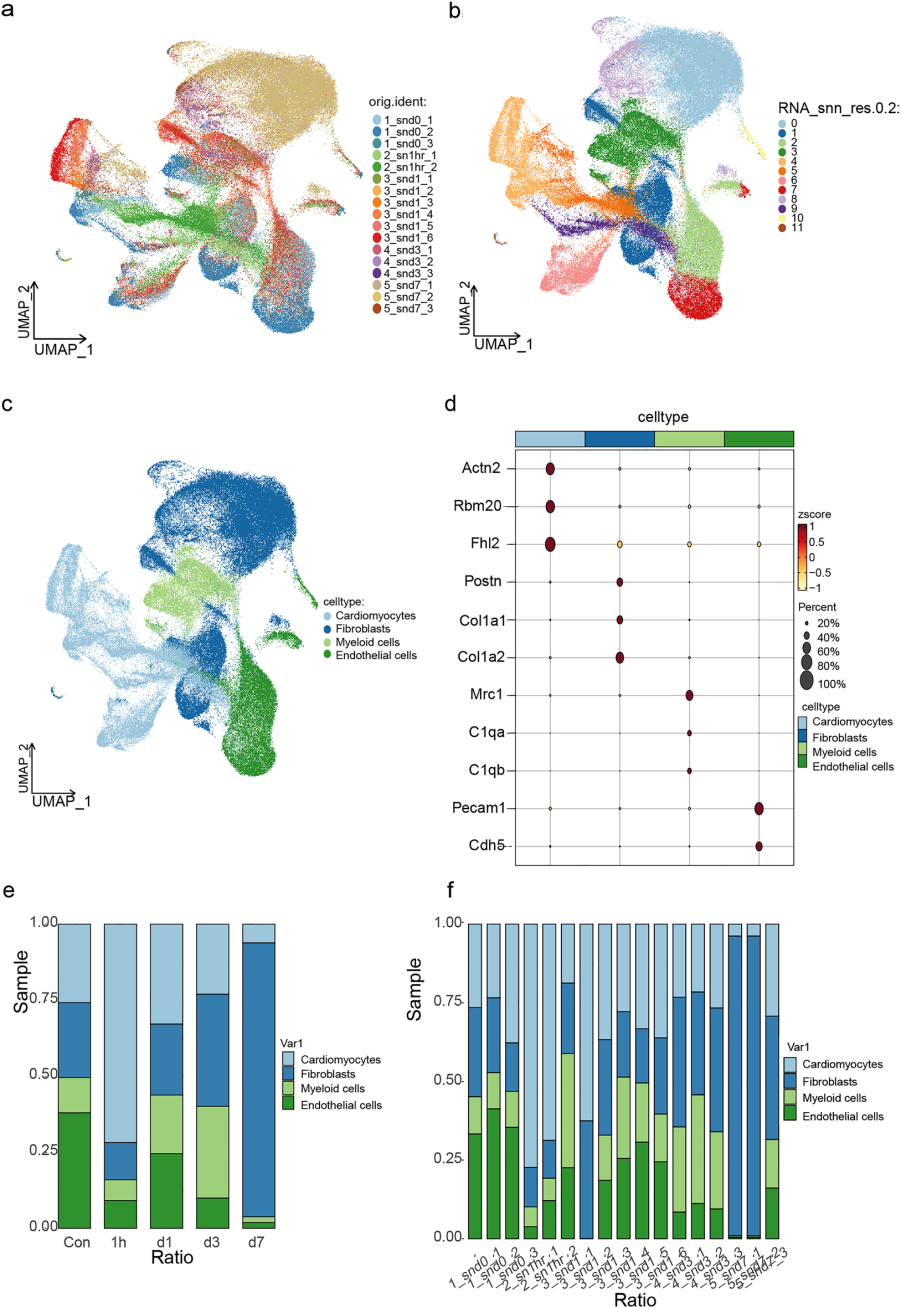
snRNA-seq data integration and clustering. (**a**) UMAP plot of all the single nuclei cells, with each color coded for a different sample. (**b**) UMAP of integrated data identified 11 cell clusters (resolution ratio = 0.2). (**c**) UMAP of all cardiac cells including four cell types. Such as, cardiomyocytes, endothelial, fibroblast, and myeloid cells. Colored by cell type. (**d**) Bubble plot showing the marker genes of each cell type. (**e**) Bar plot showed the cell proportion among different groups. (**f**) Bar plot showed the cell proportion among different samples.

snRNA-seq data from 17 mice hearts, including sham (n = 3), MI 1 h (n = 2), MI 1 d (n = 6), MI 3 d (n = 3), and MI 7 d (n = 3) heart samples^21^, were integrated by CCA for a follow-up analysis. After quality control and filtration, 162 047 cells were obtained (Supplementary Fig. S1). After defining principal components (nPCs = 20) and resolution (resolution = 0.2), a total of 11 cell clusters were identified by performing non-linear dimensionality reduction using the uniform manifold approximation and projection (UMAP) method (Fig. 1a,b). Besides, four cell types from 17 hearts were eventually separated based on specific markers (Fig. 1c). CMs exhibited a significant expression of marker genes commonly associated with cardiomyopathy^22–24^, including *Actn2*, *Rbm20*, and *Fhl2* markers. Fibroblasts were identified by the presence of markers like *Postn*, *Col1a1*, and *Col1a2* markers that were closely linked to cardiac fibrosis^25^. Myeloid cells were characterized by the expression of *Mrc1*, *C1qa*, and *C1qb* markers that were associated with immune response^26^. Endothelial cells were identified by the presence of *Pecam1* and *Cdh5* markers that maintain the vascular permeability barrier of the endothelial cell junction^27^ (Fig. 1d). Concurrently, the proportion of CMs and endothelial cells decreased, while the proportion of fibroblasts and myeloid cells relatively increased as MI progressed, which was consistent with the conventional understanding that cardiac fibrosis gradually intensified with the increased duration of MI. Notably, individual outliers in the data might be attributed to variations between samples (Fig. 1e,f).

### snRNA-seq revealed the heterogeneity and subclusters of MI CMs

**Figure 2:**
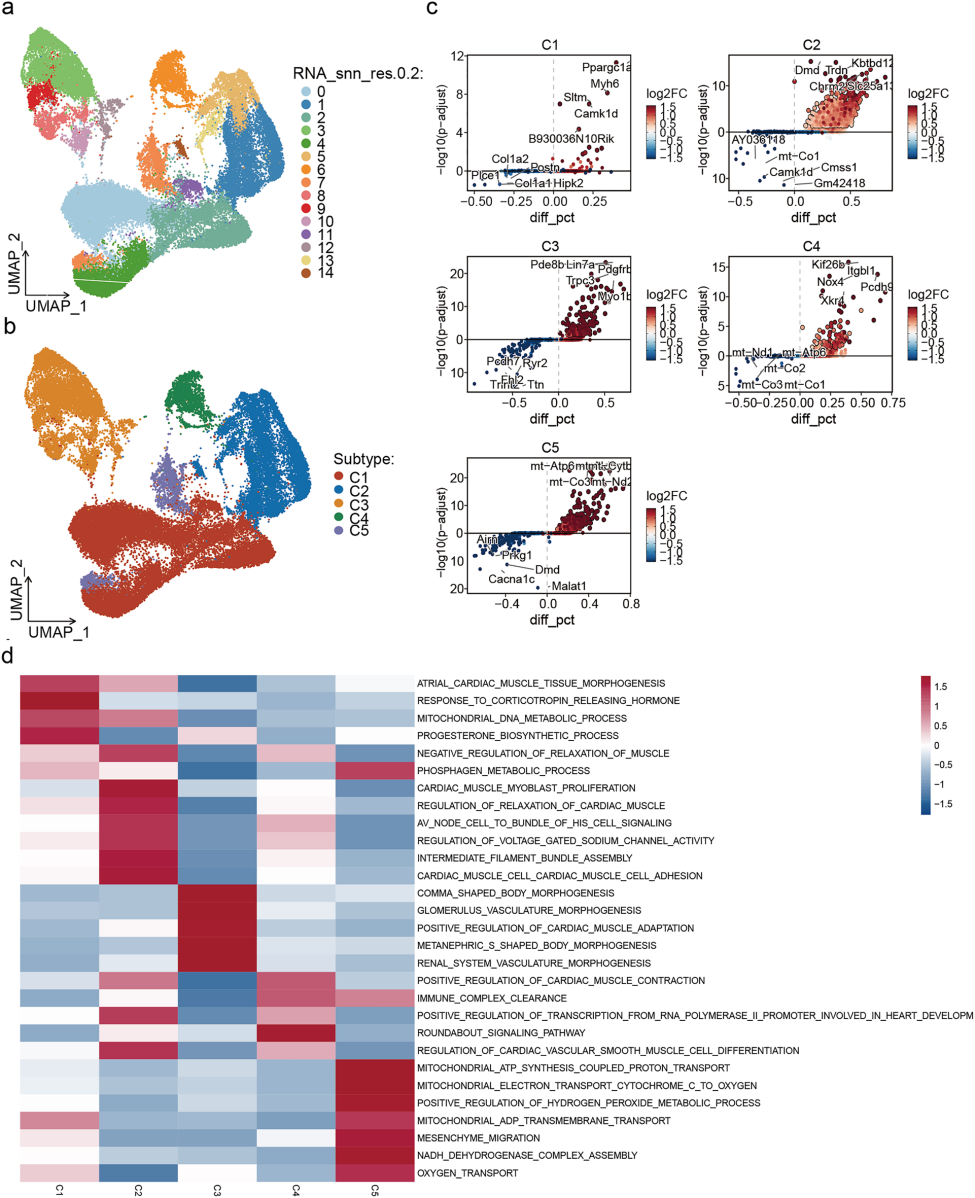
snRNA-seq revealed the heterogeneity and subclusters of MI CMs. (**a**) UMAP of integrated data identified 14 CM clusters (resolution ratio = 0.2). (**b**) Different CM subclusters identified on the UMAP. (**c**) The key genes of different CM subclusters identified on the UMAP. (**d**) Hallmark and Reactome pathways of CM subclusters determined by GSVA.

To investigate the gene expression profile underlying the heterogeneity of CMs under hypoxic conditions, our study specifically examined CMs derived from the sham and MI heart. Total CMs were clustered into 14 clusters by performing non-linear dimensionality reduction using UMAP analysis (Fig. 2a). Subsequently, 5 unique subclusters (C1–C5) were identified based on gene expression similarity (Fig. 2b). Each subcluster exhibited marker genes that characterized their distinct cell type. For instance, C1 showed high expression levels of *PGC1α*, *Myh6*, and *Camk1d*. C2 demonstrated high expression levels of *Dmd*, *Trdn*, *Kbtbd12*, *Chrm2*, and *Slc25a13*. C3 displayed increased expression levels of *Pde8b*, *Lin7a*, *Pdgfrb*, *Trpc3*, and *Myo1b*. C4 exhibited elevated expression levels of *Kif26b*, *Itgbl1*, *Nox4*, *Pcdh9*, and *Xkr4*. C5 showed elevated expression levels of mitochondrial transcripts, such as *mt*-*Atp6*, *mt*-*Cytb*, *mt*-*Co3*, and *mt-Nd2*, indicating a notable mitochondrial malfunction that could trigger CM apoptosis during this stage^28–31^ (Fig. 2c). Therefore, the UMAPs, which included both control and MI CMs, indicated that the highly expressed genes enriched in C5 of MI were potentially specific to MI.

We then conducted GSVA to investigate the possibly biological functions of the 5 CM subclusters. The results demonstrated that different CM subclusters were enriched in distinct biological processes (Fig. 2d). For example, the specific genes enriched in C1 were primarily associated with atrial cardiac muscle tissue morphogenesis, the response to corticotropin releasing hormone, and mitochondrial DNA metabolic process. It is possible that C1 was also involved in the repair of myocardial tissue injury. On the other hand, the specific genes enriched in C2 were predominantly correlated with the cardiac muscle myoblast proliferation, AV node cell to bundle of HIS cell signaling, regulation of voltage gated sodium channel activity, and cardiac muscle cell adhesion, indicating that C2 was possibly involved in cardiac electrical conduction and fibrosis. Furthermore, the specific genes enriched in C3 were mainly associated with the positive regulation of cardiac muscle adaption, suggesting indicating a potential relationship between C3 and post myocardial injury repair. Similarly, the specific genes enriched in C4 were principally correlated with positive regulation of cardiac muscle contraction, immune complex clearance, and regulation of cardiac vascular smooth muscle cell differentiation, indicating that C4 was plausible related to physical function of CMs. Lastly, the specific genes enriched in C5 were predominantly associated with the phosphagen metabolic process, mitochondrial ATP synthesis coupled protein transport, mitochondrial electron transport cytochrome C to oxygen, mitochondrial ADP transmembrane transport, NADH dehydrogenase complex assembly, and oxygen transport, indicating a close correlation between C5 and mitochondrial function, further investigation on this CM subcluster may prove beneficial in understanding the mechanisms of MI and exploring novel therapeutic targets.

### C5 was the main subcluster related to mitochondrial malfunctional and cell apoptosis after MI

**Figure 3:**
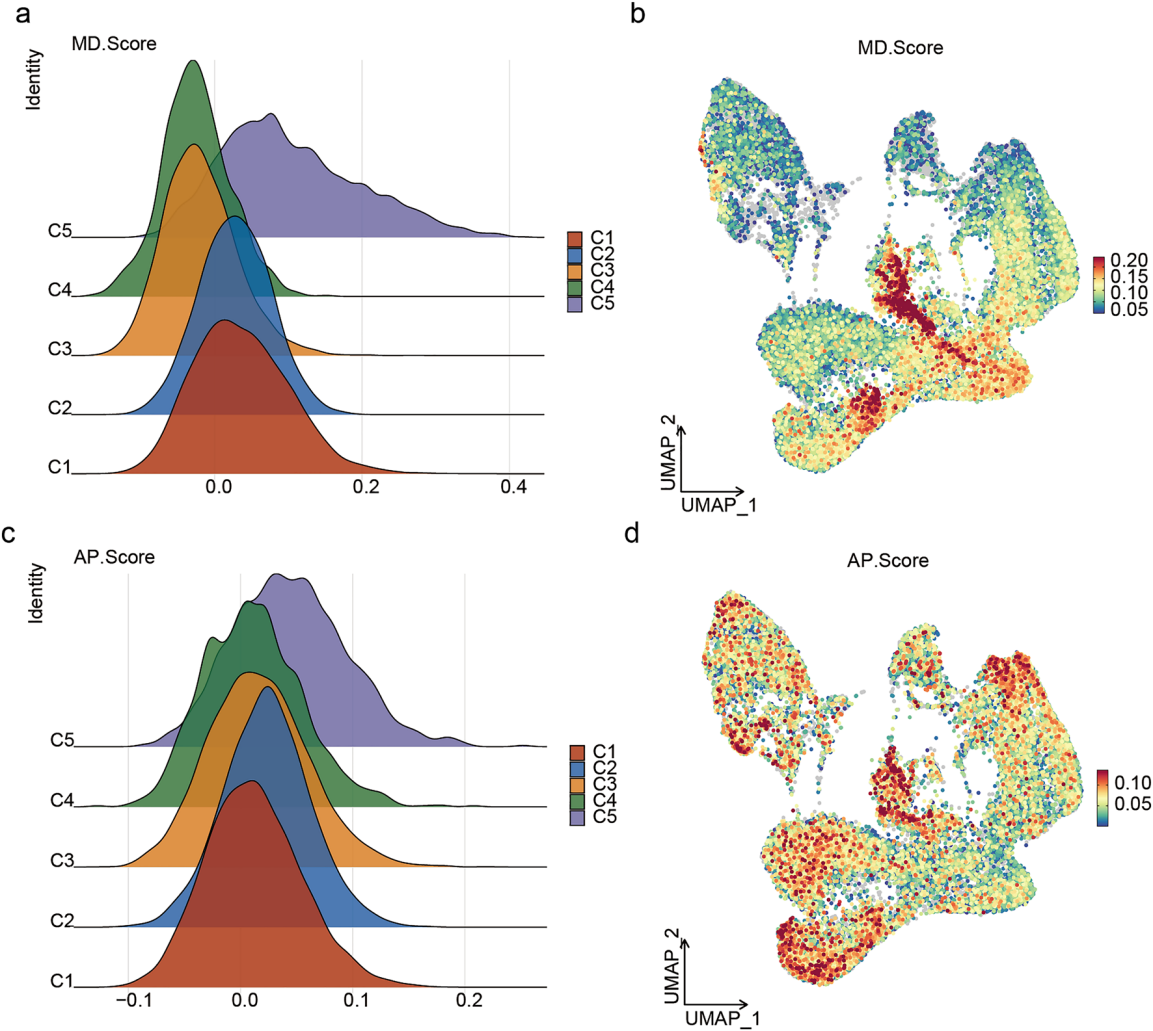
Identification the MD. Score and AP. Score of C1–C5. (**a**) The MD. Score was calculated by the Seurat function AddModuleScore. (**b**) Distribution of C1–C5 subclusters based on the MD. Score. (**c**) The AP. Score was calculated by the Seurat function AddModuleScore. (**d**) Distribution of C1–C5 subclusters based on the AP. Score.

To investigate the cellular subgroups associated with functional deterioration and the plausible pathological mechanisms leading to cardiac dysfunction, the Seurat function AddModuleScore using 3 separate mitochondrial malfunction groups and 1 apoptosis group showed that C5 exhibited a significantly higher MD. Score for mitochondrial dysfunction (Fig. 3a,b) and a markedly higher AP. Score for cell apoptosis in comparison to C1–C4 subclusters (Fig. 3c,d), indicating that C5 might be the primary subcluster responsible for ventricular remodeling caused by mitochondrial malfunction and cell apoptosis.

### Trajectory of CM subcluster remodeling after MI

**Figure 4:**
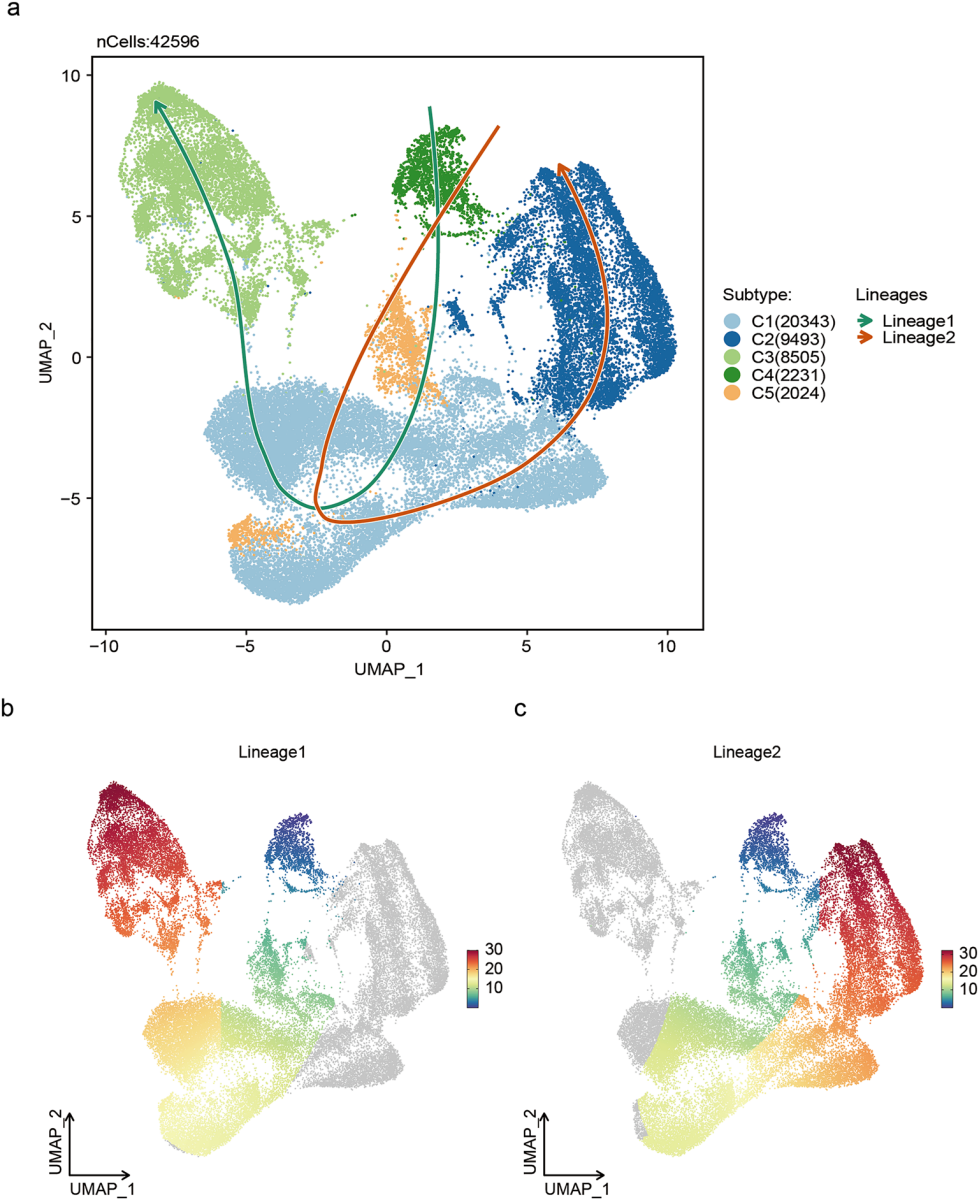
Trajectory of CM subcluster remodeling after MI. (**a**) Pseudotime trajectory analysis displayed two lineages among C1–C5 subclusters as the duration of MI escalated. (**b**) Lineage 1 of CM subclusters: C4–C5 to C1–C3. (**c**) Lineage 2 of CM subclusters: C4–C5 to C1–C2.

To explore the transition among these CM subclusters, a pseudotime trajectory analysis was conducted on all CM subclusters. The results demonstrated that CMs were separated into distinct lineages by positioning normal CMs (C4) at the initiation point of the trajectory, as the duration of MI escalated (Fig. 4a). Meanwhile, it was observed that the endpoints of pseudotime trajectories, namely C2 and C3, represented distinct end states of CMs. In light of this, two primary transition lineages were identified based on the properties of various subclusters of CMs (Fig. 4b,c).

### GO and KEGG analysis of C1–C5 subclusters

**Figure 5:**
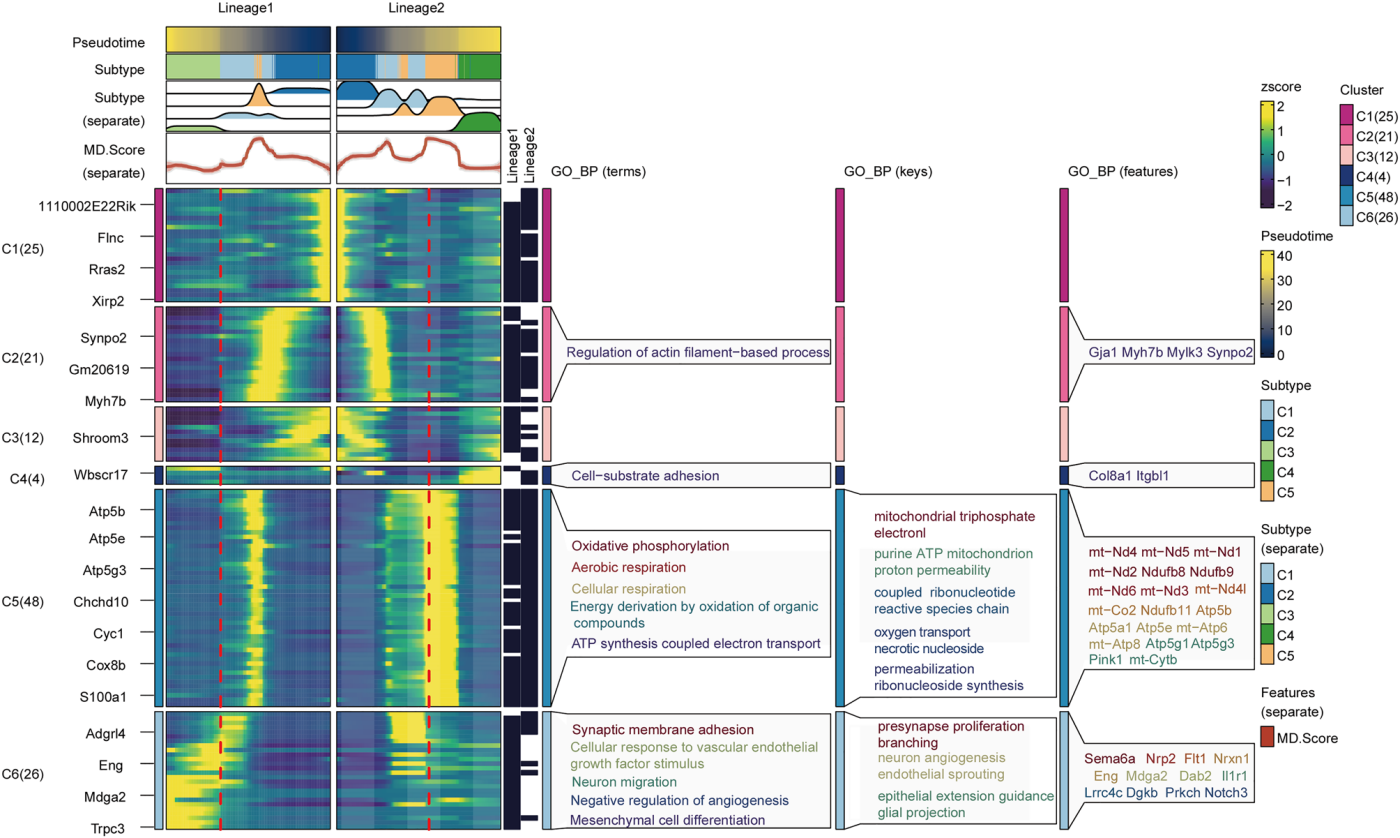
GO analysis of C1–C5 subclusters.

To better understand the transcriptional dynamics that occurred during the trajectories of CM remodeling, we simulated functional changes between C1–C5 subclusters of CMs by GO analysis based on the previously identified two lineages. The C4 subcluster in both lineages expressed specific genes such as *Col8a1* and *Itgbl1*, which were primarily involved in cell-substrate adhesion. Interestingly, a set of genes (C5 cluster) and C5 cell subcluster exhibited significant consistency and specificity, with GO biological processes and functions focusing on mitochondrial transcripts and mitochondrial-related genomic genes including *mt-Nd4*, *mt-Nd5*, *mt-Nd1*, *mt-Nd2, Ndufb8*, *Ndufb9*, *mt-Nd6*, *mt-Nd3*, *mt-Nd4l*, *mt-Co2*, *Ndufb11*, *Atp5b*, *Atp5a1*, *Atp5e*, *mt-Atp6*, *mt-Atp8*, *Atp5g1*, *Atp5g3*, *Pink1*, and *mt-Cytb*. Meanwhile, C5 cell subcluster demonstrated GO terms related to oxidative phosphorylation, aerobic respiration, cellular respiration, energy derivation by oxidation of organic compounds, and ATP synthesis coupled electron transport. Concomitantly, these genes were predominantly enriched in pathways related to mitochondrial triphosphate electron, purine ATP mitochondrion proton permeability, coupled ribonucleotide reactive species chain, oxygen transport necrotic nucleoside, and permeabilization ribonucleoside synthesis (Fig. 5).

**Figure 6:**
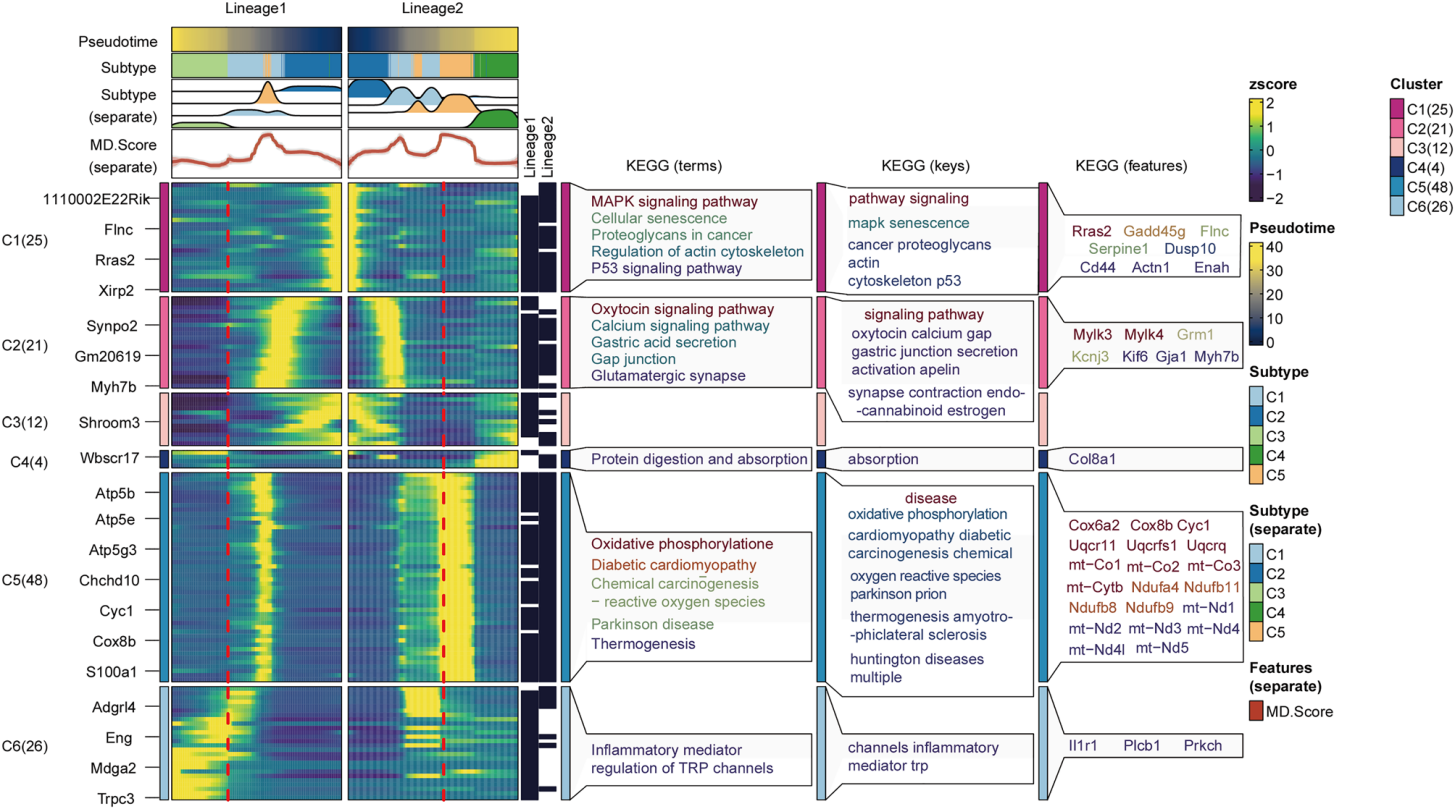
KEGG analysis of C1–C5 subclusters.

In addition, we simulated functional changes between C1–C5 clusters of CMs by KEGG analysis as well. The C4 subcluster in both lineages includes specific genes such as *Col8a1*, which were primarily involved in protein absorption. While the C5 subcluster exhibited markable consistency and specificity with C5 gene cluster as well, with KEGG pathological processes focusing on mitochondrial transcripts and mitochondrial-related genomic genes including *Cox6a2*, *Cox8b*, *Cyc1*, *Uqcr11*, *Uqcrfs1*, *Uqcrq*, *mt-Co1*, *mt-Co2*, *mt-Co3*, *mt-Cytb*, *Ndufa4*, *Ndufb11*, *Ndufb8*, *Ndufb9*, *mt-Nd1*, *mt-Nd2*, *mt-Nd3*, *mt-Nd4*, *mt-Nd4I*, and *mt-Nd5*. Concomitantly, C5 cell subcluster showed KEGG terms associated with oxidative phosphorylation, diabetic cardiomyopathy, chemical carcinogenesis, and thermogenesis diseases (Fig. 6). These findings were in line with the result that C5 exhibited the most significant MD. Score and AP. Score, aiding in identifying pathological mechanisms associated with the CM mitochondrial malfunction and apoptosis by exploring the gene expression profile of C5.

### The cell–cell communication between C1–C5 subclusters and other cell types

**Figure 7:**
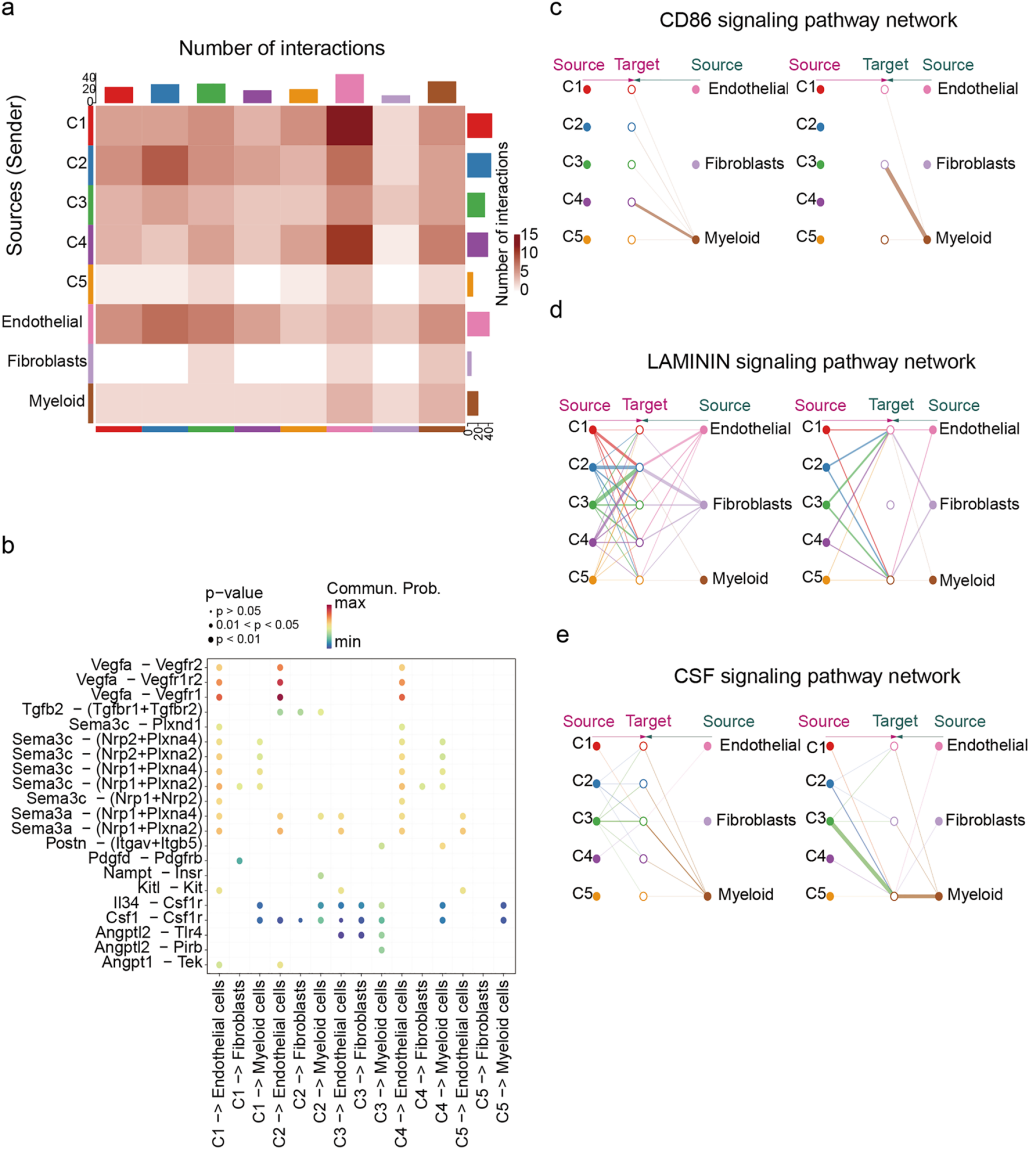
The cell–cell communication between C1–C5 subclusters and other cell types. (**a**) Heatmap showing the cell–cell communication between C1–C5 subclusters and other cell types. (**b**) Bubble plot showing the ligands and receptors of VEGF, Sema3c, Postn, TGF-β, and CSF pathways. (**c**–**e**) Cell communications demonstrating the interactions of CD86, LAMININ, and CSF signaling pathways. The thickness of the line indicated numbers of signaling targeting C1–C5 subclusters or other cells types.

In order to investigate the interactions between C1–C5 and other cell types, such as endothelial cells, smooth muscle cells, and myeloid cells, we conducted cell–cell communication analysis. The results revealed a close communication between C1, C2, C4, and endothelial cells, suggesting that C1, C2, and C4 likely belonged to subclusters that promoted angiogenesis. While C5 exhibited limited communication with other cells, indicating that C5 might lost its ability to interact with other cells due to mitochondrial malfunction and apoptosis (Fig. 7a). We further exampled the receptor-ligand interactions of various pathways. Our observations revealed that C1, C2, and C4 potentially influenced the VEGFR of endothelial cells. While C5 exhibited minimal communication with other cells as well (Fig. 7b), Additionally, our findings indicated that C4 interacted with myeloid cells in the CD86 signaling pathway, which aligned with the immune complex clearance function of C4 (Fig. 7c). C2 and C3 enhanced signal levels in the laminin pathway due to their interaction with fibroblasts, which corresponded to their position at the end of the trajectories and suggested their potential pro-fibrotic effects. Meanwhile, C1, C2, and C4 actively interacted with endothelial cells in the laminin pathway, indicating that C1, C2, and C4 might have a higher potential to promote angiogenesis (Fig. 7d). Besides, C5 displayed little communication with other cells in both the CD86 and CSF signaling pathway (Fig. 7c,e). These findings indicate that C1–C4 have close communication with other cells, while C5 exhibits limited communication with other cells due to its potentially dysfunctional state.

### Slc25a4 was increased in CMs under hypoxic conditions

**Figure 8:**
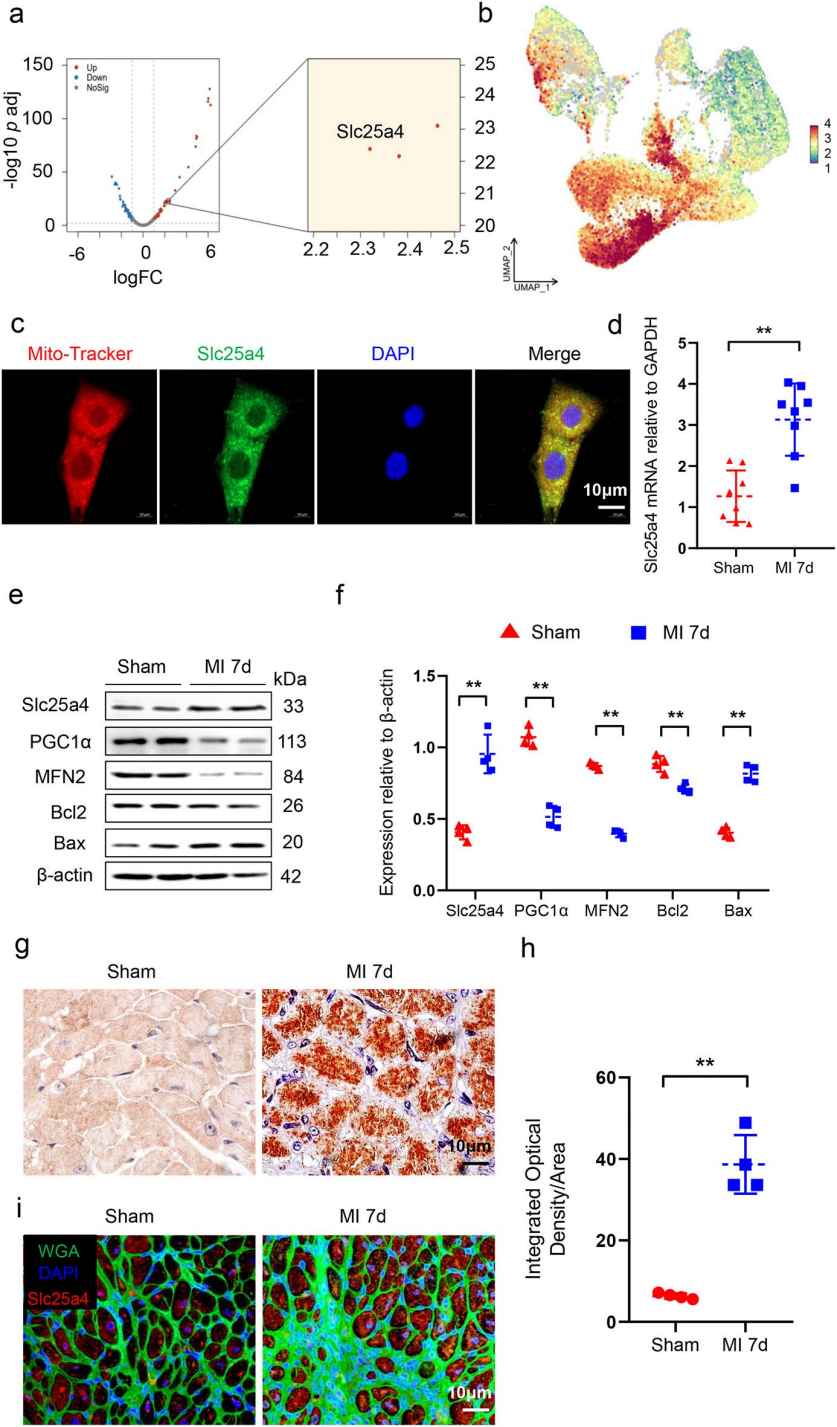
Slc25a4 was increased in CMs under hypoxic conditions. (**a**) DEA between C4 and C5 subclusters. (**b**) The expression levels of Slc25a4 in C1–C5 subclusters. (**c**) Immunofluorescence staining of Mito-Tracker and Slc25a4 in CMs. Scale bar = 10 μm. (**d**) Transcript levels of Slc25a4 in the hearts of sham and MI mice (n = 8). (**e**,**f**) Western blotting analysis and protein levels of Slc25a4 in the hearts of sham and MI mice (n = 4). (**g**,**h**) Representative immunohistochemical images with quantitation of Slc25a4 in the hearts of sham and MI mice (n = 4). Scale bar = 10 μm. (**i**) Representative immunofluorescence images of Slc25a4 in the hearts of sham and MI mice. Scale bar = 10 μm. ** P < 0.01.

A set of genes showing increased expression levels in the C5 subcluster were identified after conducting DEA between the C4 subcluster and C5 subcluster, among which Slc25a4 was upregulated in C5 and reported to correlated with mitochondrial malfunction and cell apoptosis^32,33^ (Fig. 8a,b). The mitochondrial permeability transition pore (MPTP), composed of the Slc25a4 protein, is a large channel located on the inner membrane of mitochondria. When activated, the MPTP leads to a significant increase in osmotic pressure on the inner mitochondrial membrane, causing mitochondrial swelling, membrane rupture, and ultimately resulting in mitochondrial apoptosis during ischemic injury^34,35^. In our study, immunofluorescence staining demonstrated the co-localization of Slc25a4 with mitochondria in mice cardiomyocyte line (HL-1) (Fig. 8c). To further validate the effect of Slc25a4 on mitochondrial malfunction and apoptosis in hypoxic CMs, 10-week-old C57BL/6 wild type mice underwent sham or MI for 7 days. RT-PCR and western blotting analysis confirmed a high expression level of Slc25a4 in the hearts of MI mice compared to sham mice (Fig. 8d–f). Immunohistochemical and immunofluorescence staining for Slc25a4 in heart slices of sham and MI mice further validated these results (Fig. 8g–i), suggesting that Slc25a4 might play a crucial role in the heart's response to hypoxic stress.

### Slc25a4 promoted mitochondrial malfunction and cell apoptosis in HL-1

**Figure 9:**
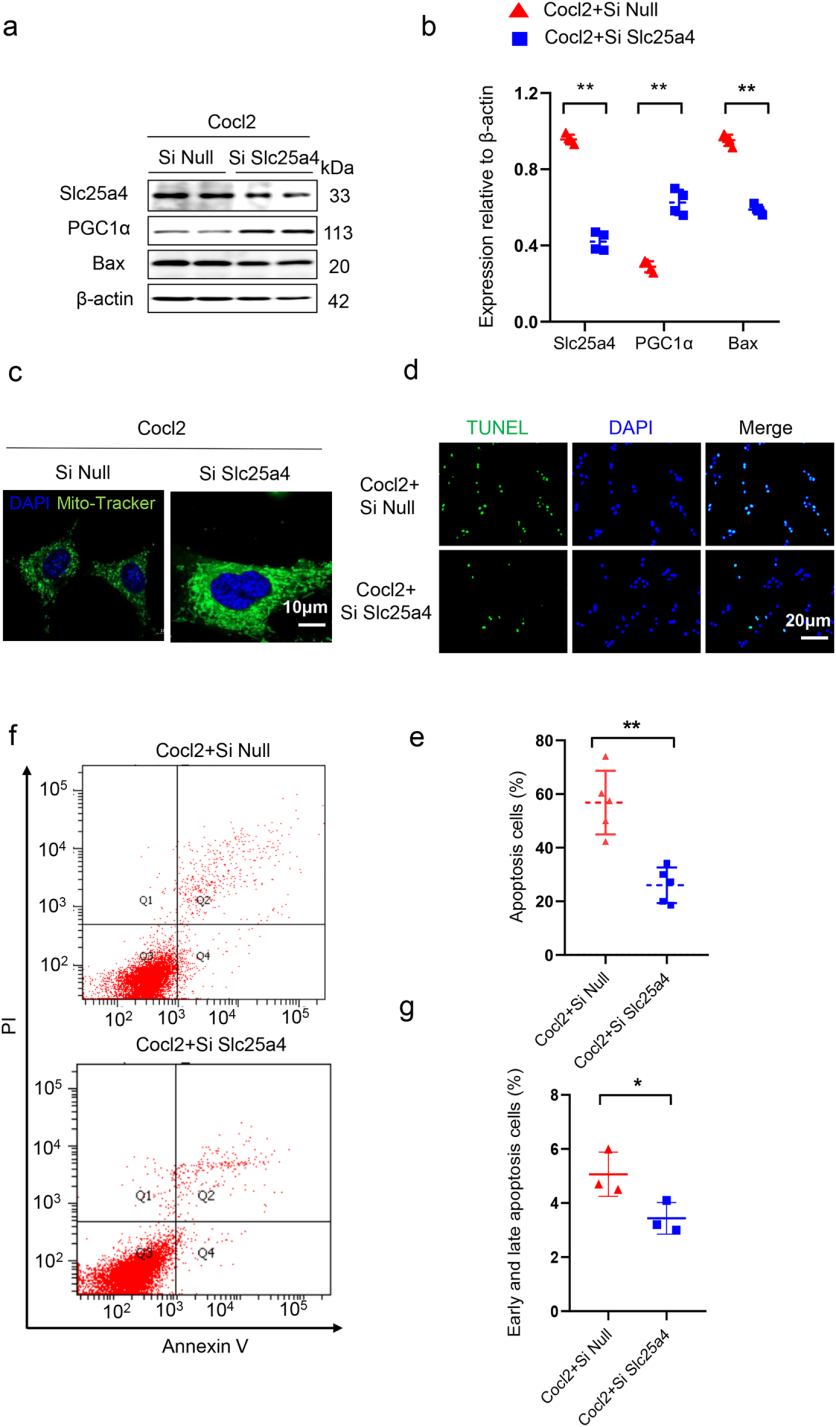
Slc25a4 promoted mitochondrial malfunction and cell apoptosis in HL-1. (**a**,**b**) Western blotting analysis and protein levels of Slc25a4 in HL-1 cells transfected with null or Slc25a4 siRNA followed by Cocl2 (400 μM) treatment for 24 h (n = 4). (**c**) Immunofluorescence staining of Mito-Tracker in HL-1 cells transfected with null or Slc25a4 siRNA followed by Cocl2 (400 μM) treatment for 24 h. Scale bar = 10 μm. (**d**,**e**) TUNEL staining of HL-1 cells transfected with null or Slc25a4 siRNA followed by Cocl2 (400 μM) treatment for 24 h. Scale bar = 20 μm. (**f**,**g**), Flow cytometry was used to test the apoptosis percentage of HL-1 cells transfected with null or Slc25a4 siRNA followed by Cocl2 (400 μM) treatment for 24 h. *P < 0.05, **P < 0.01.

To validate the function of Slc25a4 as a regulator of mitochondrial function and apoptosis, we conducted an experiment using siRNA targeting Slc25a4 to reduce its expression in HL-1 cells and using Cocl2 to trigger hypoxia in vitro. Western blotting analysis revealed that the impaired mitochondrial biogenesis and fusion as well as cell apoptosis caused by hypoxia were significantly improved after transfection with Slc25a4 siRNA (Fig. 9a,b). Mito-Tracker staining showed that Slc25a4 knockdown led to an increase in the total number of mitochondria and a reduction in the accumulation of damaged mitochondria in CMs (Fig. 9c). Additionally, the percentage of CM apoptosis under hypoxic conditions was measured using both TUNEL staining and flow cytometry analysis. Our findings indicated that Slc25a4 silence significantly reduced the percentage of CM apoptosis (Fig. 9d–g). Overall, these findings indicate that the knockdown of Slc25a4 greatly improves the mitochondrial function and cell viability of CMs under hypoxic conditions in vitro.

### Slc25a4 was increased in AC16 and promoted mitochondrial malfunction and cell apoptosis

**Figure 10:**
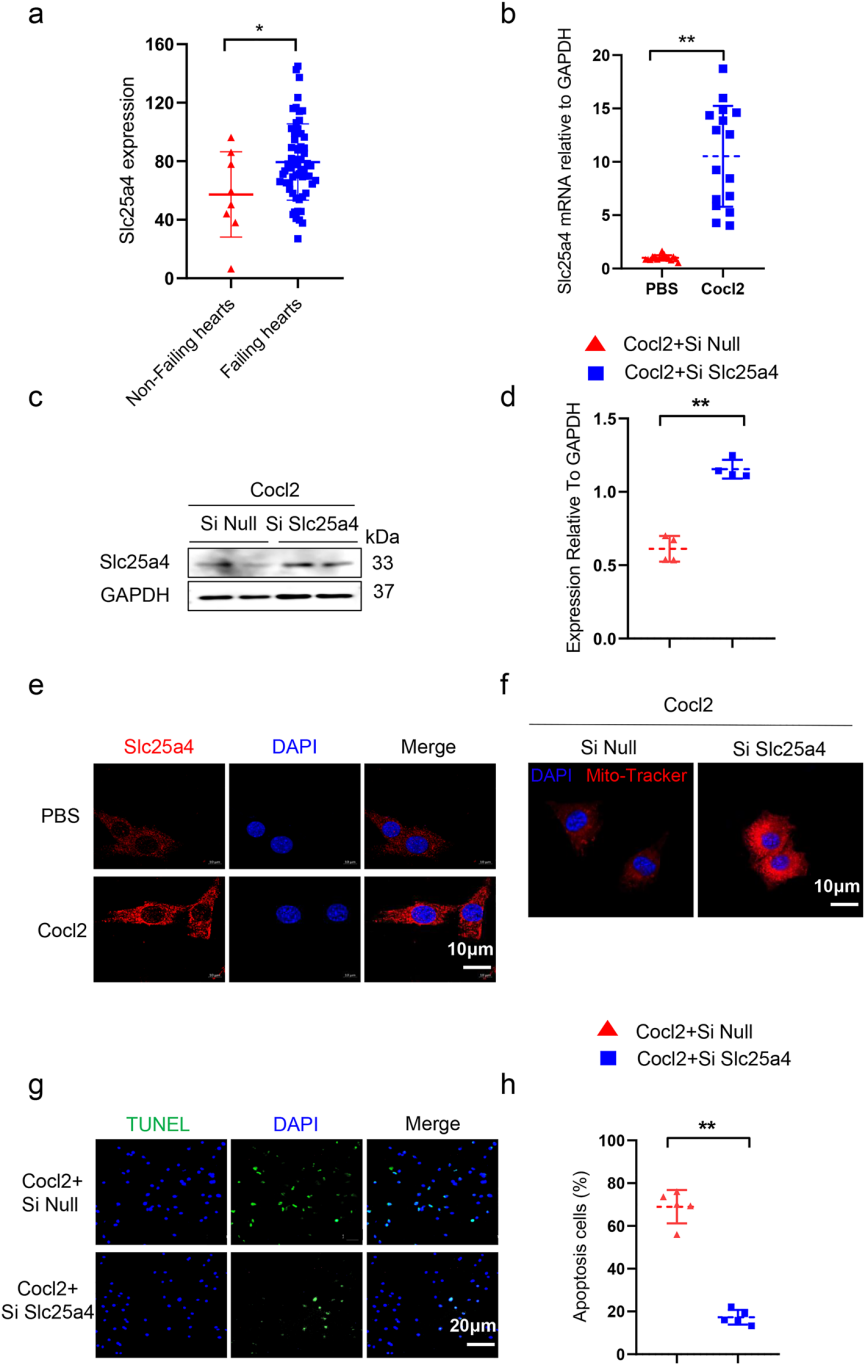
Slc25a4 promoted mitochondrial malfunction and cell apoptosis in AC16. (**a**) Transcript levels of Slc25a4 in non-failing (n = 8) and failing human hearts (n = 60) based on GSE21610 dataset. (**b**) Transcript levels of Slc25a4 in AC16 cells treated with Cocl2 for 24 h (n = 16). (**c**,**d**) Western blotting analysis and protein levels of Slc25a4 in AC16 cells treated with Cocl2 for 24 h (n = 4). (**e**) Immunofluorescence staining of Slc25a4 in AC16 cells treated with Cocl2 for 24 h. Scale bar = 10 μm. (**f**) Mito-Tracker staining in hypoxic AC16 cells with Slc25a4 silence. Scale bar = 10 μm. (**g**,**h**) TUNEL staining in hypoxic AC16 cells with Slc25a4 silence. Scale bar = 20 μm. (n = 5). *P < 0.05, **P < 0.01.

To further validate the role of Slc25a4 in regulating mitochondrial function and apoptosis in human hearts during hypoxia, the bulk RNA-seq dataset (GSE21610) was utilized. The analysis revealed a notably elevated expression of Slc25a4 in failing human hearts compared to non-failing hearts (Fig. 10a). Subsequently, human CM line (AC16) was subjected to Cocl2-induced hypoxic conditions, where various assays including RT-PCR, western blotting, and immunofluorescence demonstrated a significant upregulation of Slc25a4 in hypoxic CMs (Fig. 10b–e). Moreover, when AC16 cells were transfected with Slc25a4 siRNA prior to Cocl2 treatment, Mito-Tracker and TUNEL staining indicated an improvement in mitochondrial function and a reduction in cell apoptosis under hypoxic conditions, respectively (Fig. 10f–h). Overall, these results provide additional confirmation of the involvement of Slc25a4 in the pathogenesis of human failing hearts.

## Discussion

Recent single-cell studies have confirmed the CM heterogeneity induced by complex biological factors^9,10,34,36^. This heterogeneity poses a significant challenge in the investigation of a universal treatment for cardiac remodeling and HF following MI. While the emergence of snRNA-seq technology offers a fresh approach to studying the subclusters of CMs and their intracellular interactions.

In our study, we employed snRNA-seq to identify gene expression patterns associated with MI. By comparing the gene expression profiles of sham and MI mice hearts, we observed an enrichment of genes (*Actn2*, *Rbm20*, and *Fhl2*) in the CMs compared to other cell types. Further analysis of the genetic profile and gene correlation of CMs revealed variations in gene expression within CMs. Based on the heterogeneity, we employed a bioinformatics approach to cluster transcriptionally related cells or genes, each of which may be associated with distinct cellular functions. Moreover, by examining the scattering characteristics of CMs, we were able to link gene expression of C5 with mitochondrial malfunction and cell apoptosis, providing valuable insights into the pathological mechanisms underlying MI.

When comparing the sham and MI CMs, significant transcriptional differences could be observed between the two groups. These differences included the enrichment of well-known MI-related inflammation marker genes (*Fhl2*)^37^. Additionally, we identified genes not previously associated with MI in terms of their function, such as *Actn2* and *Rbm20*, which exhibit a correlation with numerous forms of cardiomyopathy^22,38^. The robust transcriptional induction of Actn2 and Rbm20 in hypoxic CMs was observed, which could potentially be attributed to the induction of Actn2 and Rbm20 specifically under hypoxic situations. It was currently unclear whether these transcriptional differences contributed to the pathogenesis of MI.

As CM heterogeneity is a crucial indicator of MI^39,40^, our study not only examined the heterogeneity between CMs and other distinct cell types (endothelial, fibroblast, and myeloid cells) in the hearts of MI mice, we also exhibited the presence of heterogeneous subclusters within the CMs. For example, C1 displayed the genetic characteristics of mitochondrial biogenesis, indicating a potentially highly active state of energy metabolism^41,42^. On the other hand, C5 exhibited a notable enrichment of genes linked to mitochondrial DNA, suggesting an energy metabolism imbalance and proximity to apoptosis caused by hypoxic^31,43–46^. In addition, we discovered another cluster C4. The primary function of C4 focused on positive regulation of cardiac muscle contraction, immune complex clearance, and regulation of cardiac vascular smooth muscle cell differentiation, which were commonly associated with the normal physiological function of CMs^47,48^. These findings indicated that the investigation of the pathological progression of cardiac remodeling should predominantly focus on the transition from C4 subcluster to C5 subcluster.

In addition, we established a cell–cell communication map between CMs and other cardiac cells based on receptor-ligand pairs. Our findings revealed that C1–C4 subclusters exhibited close communication with other cells, whereas C5 subcluster showed limited communication with other cells, suggesting its potential dysfunctionality. Additionally, our results demonstrated that C1, C2, and C4 subclusters exhibited strong interaction with endothelial cells, indicating their pro-angiogenesis effect. Furthermore, C2 and C3 subclusters exhibited high interaction with fibroblasts, suggesting their pro-fibrotic effect.

Our research findings indicated that Slc25a4 demonstrated a significant upregulation in C5 subcluster. Moreover, it played a critical role in the regulation of mitochondrial malfunction and cell apoptosis under hypoxic conditions, which were known to have a profound impact on the survival and physiological function of CMs^41,49^. Slc25a4 is a transport protein that plays a crucial role in connecting the production and consumption of energy on the inner membrane of mitochondria. Concurrently, Slc25a4 is closely associated with the MPTP, which can trigger cell apoptosis upon opening. Therefore, Slc25a4 is considered a dual-functional protein that not only facilitates energy transport but also induces apoptosis. The inactivation of MPTP by targeting Slc25a4 safeguards against cell death, which signifies that Slc25a4 is responsible for facilitating the apoptosis caused by MPTP^32^. In addition, the inhibition of mitochondrial swelling in CMs can potentially be linked to the prevention of the binding of mitochondrial matrix peptidylprolyl cis trans isomerase to Slc25a4^33^. According to these investigations, we postulated that Slc25a4 could facilitate CM apoptosis via pathways linked to mitochondrial injury. In our study, we substantiated the function of Slc25a4 in MI for the first time and revealed its proclivity for inducing mitochondrial malfunction and cellular apoptosis during hypoxic circumstances. Consequently, we hypothesized that the C5 marker-Slc25a4 could serve as a promising therapeutic target for the treatment of MI.

## Conclusion

This study offered a unique perspective on comprehending the pathological progression of MI by incorporating snRNA-seq. Additionally, we identified a distinct CM subcluster (C5) linked to mitochondrial malfunction and cell apoptosis. The marker Slc25a4, specific to C5 subcluster, showed significant potential as a therapeutic target for MI.

Several limitations should be considered when interpreting the findings of our research. Firstly, due to the challenge in obtaining human hearts tissue samples, we only utilized mice heart tissue and both mice and human CM lines (HL-1 and AC16) for experimental validation in the study. However, human myocardial tissue samples remain need to be collected for further experimental validation. Secondly, the snRNA-seq dataset included a limited sample size of only 17 samples, which may potentially impact the Statistical Power. Future studies should consider using snRNA-seq datasets with larger sample sizes to confirm our findings. Thirdly, further investigations are needed to uncover the underlying mechanisms through which Slc25a4 promotes mitochondrial dysfunction and apoptosis in CMs under hypoxic conditions.

### Supplementary Information

Supplementary Information 1.

Supplementary Information 2.

Supplementary Figures.

## Data Availability

The data used to support the findings of this study are included within the manuscript or supplementary information files.
