# Supplementary material for: Single-cell transcriptomics in MI identify Slc25a4 as a new modulator of mitochondrial malfunction and apoptosis-associated cardiomyocyte subcluster
Source: Sci Rep. 2024 Apr 23;14:9274. doi: 10.1038/s41598-024-59975-8 (PMC11039722; doi:10.1038/s41598-024-59975-8)
Supplement: Supplementary file 1 — Supplementary Information 1. [file 41598_2024_59975_MOESM1_ESM.pdf]

```
if (T) {rm(list = ls())
  gc()
  set.seed(123)
  setwd('D:/R/Others/MI_Zhou/')
  suppressPackageStartupMessages(library(Seurat))
  suppressPackageStartupMessages(library(ggplot2))
  suppressPackageStartupMessages(library(clustree))
  suppressPackageStartupMessages(library(cowplot))
  suppressPackageStartupMessages(library(dplyr))
  suppressPackageStartupMessages(library(data.table))
  suppressPackageStartupMessages(library(stringr))
  suppressPackageStartupMessages(library(gplots))
  suppressPackageStartupMessages(library(ggplot2))
  suppressPackageStartupMessages(library(stringr))
  suppressPackageStartupMessages(library(ggsci))
  suppressPackageStartupMessages(library(BiocParallel))
  suppressPackageStartupMessages(library(SCP))
  suppressPackageStartupMessages(library(clusterProfiler))
  suppressPackageStartupMessages(library(org.Hs.eg.db))
  suppressPackageStartupMessages(library(RColorBrewer))
}
if (T){dir = 'Sc/'
samples=list.files(dir)
samples
sceList1 = lapply(samples,function(pro){
  folder=file.path(dir,pro)
  print(pro)
  print(folder)
  print(list.files(folder))
  sce=CreateSeuratObject(counts = Read10X(folder),
                         project =  pro )
  return(sce)
})}
sce <- merge(sceList1[[1]],sceList1[-1])
DefaultAssay(sce) <- "RNA"
dim(sce)#[1]  27998 167343
mito_genes=rownames(sce)[grep("^Mt", rownames(sce))]
mito_genes
sce=PercentageFeatureSet(sce, "^Mt", col.name = "percent_mito")
fivenum(sce@meta.data$percent_mito)
ribo_genes=rownames(sce)[grep("^Rp[sl]", rownames(sce),ignore.case = T)]
ribo_genes
sce=PercentageFeatureSet(sce, "^Rp[sl]", col.name = "percent_ribo")
fivenum(sce@meta.data$percent_ribo)
```

```
hb_genes <- rownames(sce)[grep("^Hb[^(p)]", rownames(sce),ignore.case =
T)]
hb_genes
sce=PercentageFeatureSet(sce, "^Hb[^(p)]", col.name = "percent_hb")
fivenum(sce@meta.data$percent_hb)
feats <- c("nFeature_RNA", "nCount_RNA", "percent_mito", "percent_ribo",
"percent_hb")
VlnPlot(sce, group.by = "orig.ident", features = feats, pt.size = 0,
ncol = 2) +
  NoLegend()
selected_c        <-        WhichCells(sce,        expression        =
nFeature_RNA >300&nFeature_RNA<5000)#姣忎釜缁嗚優涓  熶鍥犳〃杈?300
selected_f <- rownames(sce)[Matrix::rowSums(
  sce@assays$RNA@counts > 0 ) > 3]
sce.filt <- subset(sce, features = selected_f, cells = selected_c)
dim(sce.filt)#[1]  21904 162666
table(sce.filt@meta.data$orig.ident)
# 1_snd0_1  1_snd0_2  1_snd0_3  2_sn1hr_1  2_sn1hr_2  3_snd1_1  3_snd1_2
3_snd1_3  3_snd1_4
# 12600     19945      3163      8527      15291      2412          8
5087      6209
# 3_snd1_5  3_snd1_6  4_snd3_1  4_snd3_2  4_snd3_3  5_snd7_1  5_snd7_2
5_snd7_3
# 4827      12866      3537      4835      1798      27910      27910
5741
selected_mito <- WhichCells(sce.filt, expression = percent_mito < 1)
selected_ribo <- WhichCells(sce.filt, expression = percent_ribo > 1)
selected_hb <- WhichCells(sce.filt, expression = percent_hb < 1)
sce.filt <- subset(sce.filt, cells = selected_mito)
sce.filt <- subset(sce.filt, cells = selected_hb)
VlnPlot(sce.filt, group.by = "orig.ident", features = feats, pt.size =
0, ncol = 2) +
  NoLegend()
dim(sce.filt)#[1]  21904 162047
table(sce.filt@meta.data$orig.ident)
# 1_snd0_1  1_snd0_2  1_snd0_3  2_sn1hr_1  2_sn1hr_2  3_snd1_1  3_snd1_2
3_snd1_3  3_snd1_4  3_snd1_5  3_snd1_6
# 12513     19873      3147      8489      15222      2380          8
5050      6187      4799      12821
# 4_snd3_1  4_snd3_2  4_snd3_3  5_snd7_1  5_snd7_2  5_snd7_3
# 3508      4793      1770      27839      27839      5711
sce <- sce.filt;rm(sce.filt);gc()
###
sce <- FindVariableFeatures(sce,nfeatures = 2000)
```

```
sce <- ScaleData(sce, verbose = FALSE)
sce <- RunPCA(sce, npcs = 30, verbose = FALSE)
sce <- RunUMAP(sce, reduction = "pca", dims = 1:30)
ElbowPlot(sce)
sce <- FindNeighbors(sce, dims = 1:15)
sce <- FindClusters(sce,resolution = c(0.2,0.5),
                    algorithm = 1)
CellDimPlot(srt = sce, group.by = "RNA_snn_res.0.2",
            reduction = "UMAP", label = F,
            theme_use = "theme_blank")
CellDimPlot(srt = sce, group.by = "orig.ident",
            reduction = "UMAP", label = F,
            theme_use = "theme_blank")
sce <- NormalizeData(sce, normalization.method = "LogNormalize",
scale.factor = 10000)
genes_to_check <- c('Actn2','Rbm20','Fhl2',#Cardiomyocytes3,4,5,7,9,12
                    'Postn','Col1a1','Col1a2',#Fibroblasts 0,8,13,14
                    'Mrc1','C1qa','C1qb',#Myeloid Cells 2
                    'Pecam1','Cdh5','Vwf'#Endothelial cells 1,6,10,11
)
p_all_markers <- DotPlot(sce, features = genes_to_check,col.min=-2,
col.max=2,
                         group.by =
"RNA_snn_res.0.2",assay='RNA')+coord_flip()
p_all_markers
celltype=data.frame(ClusterID=0:11,
                    celltype='Other cells')
celltype[celltype$ClusterID %in% c( 3 ),2]='Myeloid cells'
celltype[celltype$ClusterID %in% c( 0,1,8,11 ),2]='Fibroblasts'
celltype[celltype$ClusterID %in% c( 4,5,6,9 ),2]='Cardiomyocytes'
celltype[celltype$ClusterID %in% c( 2,7,10 ),2]='Endothelial cells'
head(celltype)
celltype
table(celltype$celltype)
sce@meta.data$celltype = NA
for(i in 1:nrow(celltype)){
  sce@meta.data[which(sce@meta.data$RNA_snn_res.0.2 ==
                        celltype$ClusterID[i]),'celltype'] <-
celltype$celltype[i]}
table(sce@meta.data$celltype)
sce@meta.data$celltype <- factor(sce@meta.data$celltype,
                                 levels =
c('Cardiomyocytes','Fibroblasts',
                                           'Myeloid
```

```
cells','Endothelial cells'))
Idents(sce) <- sce@meta.data$celltype
CellDimPlot(srt = sce, group.by = "celltype",
            reduction = "UMAP", label = F,
            theme_use = "theme_blank")
genes_to_check <- c('Actn2','Rbm20','Fhl2',#Cardiomyocytes 3,4,5,7,9,12
                    'Postn','Col1a1','Col1a2',#Fibroblasts 0,8,13,14
                    'Mrc1','C1qa','C1qb',#Myeloid Cells 2
                    'Pecam1','Cdh5'#Endothelial cells 1,6,10,11
)
ht <- GroupHeatmap(
  srt = sce,
  features = genes_to_check,
  group.by = c("celltype"),
  heatmap_palette = "YlOrRd",
  cell_annotation_palette = c("Dark2", "Paired", "Paired"),
  show_row_names = F, row_names_side = "left",
  add_dot = TRUE, add_reticle = TRUE
)
print(ht$plot)
getPalette = colorRampPalette(brewer.pal(12, "Paired"))
colors.number = 60
mycolors <- getPalette(60)
show_palettes(mycolors[c(1,5,12,17,33,39,22,28)])
mycol <- mycolors[c(1,5,12,17)]
timepoint <- data.frame(
  sample=table(sce$orig.ident),

timepoint=factor(c(rep('Con',3),rep('1h',2),rep('d1',6),rep('d3',3),re
p('d7',3))))
for(i in 1:nrow(timepoint)){
  sce@meta.data[which(sce@meta.data$orig.ident ==
                        timepoint$sample.Var1[i]),'timepoint']      <-
timepoint$timepoint[i]}
sce$timepoint        <-        factor(sce$timepoint,levels        =
c('Con','1h','d1','d3','d7'))
table(sce$timepoint)
timepoint <- prop.table(table(sce$celltype,sce$timepoint), margin = 2)
timepoint <- as.data.frame(timepoint)
ggplot(timepoint) +
  geom_bar(aes(x=Freq, y=Var2, fill=Var1), stat="identity", width=0.7,
size=0.5, colour='black')+
  theme(panel.background = element_blank(),
        axis.text.x = element_text()) +
```

```
  labs(x='Sample', y='Ratio') +
  coord_flip() +
  theme(panel.border = element_rect(fill=NA, color="black", size=0, linetype=0)) +
  scale_fill_manual(values=mycol)
Cellratio <- prop.table(table(sce$celltype,sce$orig.ident), margin = 2)
Cellratio <- as.data.frame(Cellratio)
ggplot(Cellratio) +
  geom_bar(aes(x=Freq, y=Var2, fill=Var1), stat="identity", width=0.7, size=0.5, colour='black')+
  theme(panel.background = element_blank(),
        axis.text.x = element_text(angle = 30, hjust = 1)) +
  labs(x='Sample', y='Ratio') +
  coord_flip() +
  theme(panel.border = element_rect(fill=NA, color="black", size=0, linetype=0)) +
  scale_fill_manual(values=mycol)

sce.C <- sce[,sce@meta.data$celltype%in%c('Cardiomyocytes')]
DefaultAssay(sce.C) <- 'RNA'
sce.C <- FindVariableFeatures(sce.C, selection.method = "vst", nfeatures = 2000)
top10 <- head(VariableFeatures(sce.C), 10)
VariableFeaturePlot(sce.C)
all.genes <- rownames(sce.C)
sce.C <- ScaleData(sce.C, features = all.genes)
sce.C <- RunPCA(sce.C, features = VariableFeatures(object = sce.C))
print(sce.C[["pca"]], dims = 1:5, nfeatures = 5)
VizDimLoadings(sce.C, dims = 1:2, reduction = "pca")
DimPlot(sce.C, reduction = "pca")
DimHeatmap(sce.C, dims = 1:15, cells = 500, balanced = TRUE)
ElbowPlot(sce.C)
sce.C <- FindNeighbors(sce.C, dims = 1:15)
sce.C <- FindClusters(sce.C, resolution = c(0.2,0.5))
head(Idents(sce.C), 5)
sce.C <- RunUMAP(sce.C, dims = 1:15)
CellDimPlot(srt = sce.C, group.by = "RNA_snn_res.0.2",
            reduction = "UMAP", label = T,pt.size = 0.6,
            theme_use = "theme_blank")
Idents(sce.C) <- sce.C@meta.data$RNA_snn_res.0.2
sce.markers <- FindAllMarkers(object = sce.C, only.pos = TRUE,
                              min.pct = 0.25,
                              thresh.use = 0.25)
top50 <- sce.markers %>% group_by(cluster) %>% top_n(50, avg_log2FC)
```

```
write.csv(top50,'top50.Cardiomyocytes.csv')
celltype=data.frame(ClusterID=0:14,
                    celltype='Ungrouped')
celltype[celltype$ClusterID %in% c(0,2,4,11),2]='C1'
celltype[celltype$ClusterID %in% c(1,5,13,14),2]='C2'
celltype[celltype$ClusterID %in% c(3,8,9,10,12),2]='C3'
celltype[celltype$ClusterID %in% c(6),2]='C4'
celltype[celltype$ClusterID %in% c(7),2]='C5'
sce.C@meta.data$Subtype='NA'
for (i in 1:nrow(celltype)) {

sce.C@meta.data[which(sce.C@meta.data$RNA_snn_res.0.2==celltype$Cluste
rID[i]),'Subtype'] <- celltype$celltype[i]}
sce.C@meta.data$Subtype <- factor(sce.C@meta.data$Subtype,
                                   levels                              =
c('C1','C2','C3','C4','C5'))
CellDimPlot(srt = sce.C, group.by = "Subtype",palette = 'nejm',
            reduction = "UMAP", label = F,pt.size = 0.5,
            theme_use = "theme_blank")
mycolors <- getPalette(60)
show_palettes(pal_nejm("default",alpha = 0.8)(8))
mycol <- pal_nejm("default",alpha = 0.8)(5)
Idents(sce.C) <- 'Subtype'
sce1 <- subset(sce.C,downsample=50)
sce1 <- RunDEtest(srt = sce1, group_by = "Subtype",
                  fc.threshold = 1, only.pos = FALSE)
VolcanoPlot(srt = sce1, group_by = "Subtype",ncol = 2)
DEGs <- sce1@tools$DEtest_Subtype$AllMarkers_wilcox
DEGs <- DEGs[with(DEGs, avg_log2FC > 1 & p_val_adj < 0.05), ]
write.csv(DEGs,'DEGs.csv')
Cellratio <- prop.table(table(sce.C$Subtype,sce.C$orig.ident), margin =
2)
Cellratio <- as.data.frame(Cellratio)
ggplot(Cellratio) +
  geom_bar(aes(x=Freq, y=Var2, fill=Var1), stat="identity", width=0.7,
size=0.5, colour='black')+
  theme(panel.background = element_blank(),
        axis.text.x = element_text(angle = 30, hjust = 1)) +
  labs(x='Sample', y='Ratio') +
  coord_flip() +
  theme(panel.border  =  element_rect(fill=NA,  color="black",  size=0,
linetype=0)) +
  scale_fill_manual(values=mycol)
Cellratio <- prop.table(table(sce.C$Subtype,sce.C$timepoint), margin =
```

```
2)
Cellratio <- as.data.frame(Cellratio)
ggplot(Cellratio) +
  geom_bar(aes(x=Freq, y=Var2, fill=Var1), stat="identity", width=0.7,
size=0.5, colour='black')+
  theme(panel.background = element_blank(),
        axis.text.x = element_text(angle = 30, hjust = 1)) +
  labs(x='Sample', y='Ratio') +
  coord_flip() +
  theme(panel.border = element_rect(fill=NA, color="black", size=0,
linetype=0)) +
  scale_fill_manual(values=mycol)

Idents(sce.C) <- 'Subtype'
sce.C2 <- subset(sce.C,downsample=500)
av <- as.matrix(sce.C2@assays$RNA@counts)
colnames(av) <- sce.C2@meta.data$Subtype
library(msigdbr)
library(clusterProfiler)
library(org.Hs.eg.db)
library(GSVA)
library(GSEABase)
library(pheatmap)
KEGG <- msigdbr(species = "Homo sapiens", category = "C2", subcategory
= "CP:KEGG")
KEGG$gs_name <- substr(KEGG$gs_name,6,100)
GOBP <- msigdbr(species = "Homo sapiens", category = "C5", subcategory
= "GO:BP")
GOBP$gs_name <- substr(GOBP$gs_name,6,100)
all_genes_sets <- rbind(GOBP,KEGG)
gs <- split(all_genes_sets$gene_symbol,all_genes_sets$gs_name)
gs <- lapply(gs, unique)
gsc <- GeneSetCollection(mapply(function(geneIds,keggId){
  GeneSet(geneIds,geneIdType=EntrezIdentifier(),
          collectionType=KEGGCollection(keggId),
          setName=keggId)
},gs,names(gs)))
rownames(av) <- toupper(rownames(av))
es.max <- gsva(av,gsc,parallel.sz=12)
GSVA_res <- es.max
GSVA_res <- data.frame(1:nrow(es.max),row.names = rownames(es.max))
for (i in 1:5) {
  x <- paste0('C',i)
y <- apply(es.max[,colnames(es.max)==x],1,mean)
```

```
GSVA_res[,i] <- y
colnames(GSVA_res)[i] <- x
}
library(limma)
table(sce.C2@meta.data$Subtype)
group <- factor(c('C','C','C','T','C'),levels = c('T','C'))
design <- model.matrix(~0+factor(group))
colnames(design) <- levels(factor(group))
rownames(design) <- colnames(GSVA_res)
contrast.matrix<-makeContrasts(T-C,levels = design)
fit <- lmFit(GSVA_res,design)
fit2 <- contrasts.fit(fit, contrast.matrix)
fit2 <- eBayes(fit2)
diff4 = topTable(fit2, coef=1, n=Inf)
diff4 <- diff4[order(diff4$logFC,decreasing = T),]
View(diff4)
Sig <- c(
  #E1

'ATRIAL_CARDIAC_MUSCLE_TISSUE_MORPHOGENESIS','RESPONSE_TO_CORTICOTROPI
N_RELEASING_HORMONE',

'MITOCHONDRIAL_DNA_METABOLIC_PROCESS','PROGESTERONE_BIOSYNTHETIC_PROCE
SS',

'NEGATIVE_REGULATION_OF_RELAXATION_OF_MUSCLE','PHOSPHAGEN_METABOLIC_PR
OCESS',
  #E2

'CARDIAC_MUSCLE_MYOBLAST_PROLIFERATION','REGULATION_OF_RELAXATION_OF_C
ARDIAC_MUSCLE',

'AV_NODE_CELL_TO_BUNDLE_OF_HIS_CELL_SIGNALING','REGULATION_OF_VOLTAGE_
GATED_SODIUM_CHANNEL_ACTIVITY',

'INTERMEDIATE_FILAMENT_BUNDLE_ASSEMBLY','CARDIAC_MUSCLE_CELL_CARDIAC_M
USCLE_CELL_ADHESION',
  #E3

'COMMA_SHAPED_BODY_MORPHOGENESIS','GLOMERULUS_VASCULATURE_MORPHOGENESI
S',

'POSITIVE_REGULATION_OF_CARDIAC_MUSCLE_ADAPTATION','METANEPHRIC_S_SHAP
ED_BODY_MORPHOGENESIS',
```

```
  'RENAL_SYSTEM_VASCULATURE_MORPHOGENESIS',
  #E4

'POSITIVE_REGULATION_OF_CARDIAC_MUSCLE_CONTRACTION','IMMUNE_COMPLEX_CL
EARANCE',

'POSITIVE_REGULATION_OF_TRANSCRIPTION_FROM_RNA_POLYMERASE_II_PROMOTER_
INVOLVED_IN_HEART_DEVELOPM',
  'ROUNDABOUT_SIGNALING_PATHWAY',
  'REGULATION_OF_CARDIAC_VASCULAR_SMOOTH_MUSCLE_CELL_DIFFERENTIATION',
  #C5

'MITOCHONDRIAL_ATP_SYNTHESIS_COUPLED_PROTON_TRANSPORT','MITOCHONDRIAL_
ELECTRON_TRANSPORT_CYTOCHROME_C_TO_OXYGEN',

'POSITIVE_REGULATION_OF_HYDROGEN_PEROXIDE_METABOLIC_PROCESS','MITOCHON
DRIAL_ADP_TRANSMEMBRANE_TRANSPORT',

'MESENCHYME_MIGRATION','NADH_DEHYDROGENASE_COMPLEX_ASSEMBLY','OXYGEN_T
RANSPORT'
)
pheatmap(GSVA_res[Sig,], cluster_cols = FALSE, scale = 'row',
cluster_rows = FALSE,
         color = colorRampPalette(colors = c("#00468BFF", 'white',
"#AD002AFF"))(100),
         border_color = "black")

HP_MD <-
read.gmt('gmt/HP_MUSCLE_ABNORMALITY_RELATED_TO_MITOCHONDRIAL_DYSFUNCTI
ON.v2023.2.Hs.gmt')
WP_MD <-
read.gmt('gmt/WP_NAD_METABOLISM_IN_ONCOGENE_INDUCED_SENESCENCE_AND_MIT
OCHONDRIAL_DYSFUNCTION_ASSOCIATED_SENESCENCE.v2023.2.Hs.gmt')
RE_MD <-
read.gmt('gmt/REACTOME_MITOCHONDRIAL_BIOGENESIS.v2023.2.Hs.gmt')
MD_genes <- c(HP_MD$gene,WP_MD$gene,RE_MD$gene)
MD_genes <- MD_genes[!duplicated(MD_genes)]
MD_genes <- stringr::str_to_title(MD_genes)
MD_genes <- list(MD_genes[MD_genes%in%rownames(sce)])
sce.C <- AddModuleScore(sce.C,features = MD_genes,seed = 123,name =
'MD.Score')
colnames(sce.C@meta.data)[12] <- 'MD.Score'
FeatureDimPlot(
  srt = sce.C, features = c("MD.Score"),pt.size = 1,#,'Cdc20','Ube2c'
```

```
  compare_features = F, label = F, label_insitu = F,
  reduction = "UMAP", theme_use = "theme_blank"
)
FeatureDimPlot(
  srt = sce.C, features = c('Ube2c'),pt.size = 1,
  compare_features = F, label = F, label_insitu = F,
  reduction = "UMAP", theme_use = "theme_blank"
)
FeatureDimPlot(
  srt = sce.C, features = c('Npl','Ddah1'),pt.size = 1,
  compare_features = F, label = F, label_insitu = F,
  reduction = "UMAP", theme_use = "theme_blank"
)
cor.test(sce.C@assays$RNA@counts['Cdc20',],sce.C@assays$RNA@counts['Ub
e2c',])
RidgePlot(sce.C, features = 'MD.Score',cols = mycol)
sce.C <- RunSlingshot(sce.C,group.by = 'Subtype',reduction = 'UMAP',
                      seed = 11)
FeatureDimPlot(sce.C,features = paste0('Lineage',c(1,2)),
             reduction = 'UMAP',theme_use = 'theme_blank')
CellDimPlot(sce.C,group.by = 'Subtype',reduction = 'UMAP',
           lineages = paste0('Lineage',c(1,2)),lineages_span = 1)
###load('12.26.RData')
table(sce.C$Subtype)
sce.C1 <- subset(sce.C,downsample=100)
rm(list=c('sce.C2','sce.C','sce','sceList1'));gc()
sce.C1          <-          RunDynamicFeatures(sce.C1,lineages          =
c('Lineage1','Lineage2'),n_candidates = 200)
ht <- DynamicHeatmap(sce.C1,lineages = c('Lineage1','Lineage2'),
                     use_fitted    =    T,n_split    =    6,reverse_ht    =
c('Lineage1'),
                     species = 'Mus_musculus',db='GO_BP',anno_terms = T,
                     anno_keys = T,anno_features = T,height = 5,width =
2,
                     pseudotime_label   =   25,pseudotime_label_color   =
'red',
                     separate_annotation                              =
list('Subtype','MD.Score','AP.Score'),
                     separate_annotation_palette                      =
c('Paired','nejm','jama'),
                     heatmap_palette    =    'viridis',cell_annotation    =
'Subtype')
ht$plot
ht <- DynamicHeatmap(sce.C1,lineages = c('Lineage1','Lineage2'),
```

```
                     use_fitted   =   T,n_split   =   6,reverse_ht   =
c('Lineage1'),
                     species = 'Mus_musculus',db='KEGG',anno_terms = T,
                     anno_keys = T,anno_features = T,height = 5,width =
2,
                     pseudotime_label  =  25,pseudotime_label_color  =
'red',
                     separate_annotation                              =
list('Subtype','MD.Score','AP.Score'),
                     separate_annotation_palette                      =
c('Paired','nejm','jama'),
                     heatmap_palette   =   'viridis',cell_annotation   =
'Subtype')
ht$plot
DynamicPlot(sce.C1,c("Lineage1","Lineage2"),group.by = 'Subtype',
            point_palette = 'nejm',
            features = c('Cdc20','Ube2c','Slc25a4'),
            compare_lineages = T,
            compare_features = F)
library(DESeq2)
sce.C2 <- subset(sce.C1,Subtype%in%c('C4','C5'))
expr <- data.frame(sce.C2@assays$RNA@counts)
group <- factor(sce.C2@meta.data$Subtype,levels = c('C4','C5'))
coldata <- data.frame(row.names=colnames(expr), group)
dds <- DESeqDataSetFromMatrix(
  countData = (expr+1), colData = coldata,
  design = ~ group)
dds <- DESeq(dds)
res <- results(dds,alpha=0.05)
resdata <- as.data.frame(res)
resdata$gene <- rownames(resdata)
resdata$group    =    factor(ifelse(resdata$padj    <    0.01    &
abs(resdata$log2FoldChange) >= 1,
                              ifelse(resdata$log2FoldChange>=
1 ,'Up','Down'),
                              'NoSig'),levels=c('Up','Down','NoSig'))
table(resdata$group)
cols.names <- unique(resdata$group)
cols.code <- c('#808080','#0072B5FF',"#BC3C29FF")
names(cols.code) <- cols.names
sizes <- -log10(resdata$padj)/5
sizes[sizes=="Inf"] <- 0
names(sizes) <- resdata$gene
add.alpha <- function(col, alpha=1){
```

```
  if(missing(col))
    stop("Please provide a vector of colours.")
  apply(sapply(col, col2rgb)/255, 2,
        function(x)
          rgb(x[1], x[2], x[3], alpha=alpha))
}
gray.alpha <- add.alpha("orange", alpha=0.1)

par(mfrow=c(1,2),mar = par()$mar + c(3,0,0,3))
logFC <- resdata$log2FoldChange
lg_padj <- -log10(resdata$padj)
names(logFC) <- resdata$gene
names(lg_padj) <- resdata$gene
plot(logFC, lg_padj,
     col = cols.code[as.character(resdata$group)],
     xlim=c(-6.5,6.5),ylim=c(0,150),
     ylab="", xlab="logFoldChange",
     yaxt='n',
     pch=16,
     cex=0.65
)
axis(side = 2)
title(ylab="-log10 padj",
      mgp=c(2,1,0))
if(T){
  u <- par("usr")
  f <- c(1,2.5,5,10,20)
  s <- sqrt(f/3)
  legend("bottom",
         inset=c(0,-.5),
         legend=rep("", length(f)),
         title = "Significance level [-log10(q)]",
         pch=1, pt.cex=s, bty='n',
         horiz = TRUE,
         col="#88888888")
}
par(xpd = F)
abline(h = 2, col = "gray60", lwd = 1, lty = 2)
abline(v = 1, col = "gray60", lwd = 1, lty = 2)
abline(v = -1, col = "gray60", lwd = 1, lty = 2)

rect(2.2,20,2.5,25, col = gray.alpha, border=TRUE)
plot(logFC, lg_padj,
     col = cols.code[as.character(resdata$group)],
```

```
     xlim = c(2.2,2.5), ylim=c(20,25),
     pch = 16,
     ylab = "",xlab="",
     yaxt='n',
     cex = 1
)
axis(side = 4)
up <- which(logFC > 1.5&logFC<2.5)
up2 <- which(lg_padj<280&lg_padj>190)
up <- intersect(up,up2)
up <- 'Slc25a4'
par(xpd=TRUE)
text(logFC[up],lg_padj[up], names(logFC[up]),pos=3)

rect(par("usr")[1], par("usr")[3], par("usr")[2], par("usr")[4], col =
gray.alpha)
legend("bottom",
       inset=c(0,-.5),
       ncol = 3,
       pch=16, col=cols.code, legend=cols.names, bty="n")
recordedplot1 <- recordPlot()
write.csv(resdata,'DEGs_between_C5vsC4.csv')
pdf("vocanol_plot_pro2.pdf",10,5.5)
recordedplot1
dev.off()
FeatureDimPlot(
  srt = sce.C, features = c('Slc25a4'),pt.size = 1,
  compare_features = F, label = F, label_insitu = F,
  reduction = "UMAP", theme_use = "theme_blank"
)
library(CellChat)
Idents(sce) <- 'celltype'
table(sce@meta.data$celltype)
sce.cc <- subset(sce,downsample=500)
sce.cc <- sce.cc[,sce.cc@meta.data$celltype%in%c(
  'Endothelial cells','Myeloid cells','Fibroblasts'
)]
sce.C1 <- sce.C[,sce.C@meta.data$Subtype%in%c(
  'C1','C2','C3','C4','C5'
)]
sce.cc2 <- subset(sce.C1,downsample=500)
table(sce.cc2@meta.data$celltype)
sce.cc2@meta.data$celltype <- sce.cc2@meta.data$Subtype
sce.cc <- merge(sce.cc,sce.cc2)
```

```
rm(sce.cc2)
table(sce.cc@meta.data$celltype)
DefaultAssay(sce.cc) <- "RNA"
cellchat  <-  createCellChat(sce.cc,group.by  =  'celltype',meta  =
sce.cc@meta.data)
CellChatDB <- CellChatDB.mouse
unique(CellChatDB$interaction$annotation)
CellChatDB.use <- subsetDB(CellChatDB,
                           search = 'Secreted Signaling')###
cellchat@DB <- CellChatDB.use
cellchat <- CellChat::subsetData(cellchat)
cellchat <- identifyOverExpressedGenes(cellchat)
cellchat <- identifyOverExpressedInteractions(cellchat)
cellchat <- projectData(cellchat,PPI.mouse)
cellchat <- computeCommunProb(cellchat,raw.use = F,population.size = T)
cellchat <- computeCommunProbPathway(cellchat)
cellchat <- aggregateNet(cellchat)
cellchat <- netAnalysis_computeCentrality(cellchat,slot.name='netP')
cellchat <- filterCommunication(cellchat,min.cells = 10)
df.netp <- subsetCommunication(cellchat)
cellchat <- aggregateNet(cellchat)
table(cellchat@idents)
groupSize <- as.numeric(table(cellchat@idents))
par(mfrow=c(1,2),xpd=T)
netVisual_circle(cellchat@net$count,vertex.weight=groupSize,weight.sca
le=T,
                 label.edge=F,title.name='Number of interactions')
netVisual_circle(cellchat@net$weight,vertex.weight=groupSize,weight.sc
ale=T,
                 label.edge=F,title.name='interaction
weights/strength')

netVisual_heatmap(cellchat,title.name='Number of interactions',
                  color.heatmap = c('white','darkred'))
netVisual_hierarchy2(cellchat@net$count,vertex.receiver =c(
  2:5
) ,
weight.scale = F,
vertex.weight = 0.5,
vertex.weight.max = 10,
vertex.size.max = 10,
edge.weight.max = 10,
edge.width.max = 0.1,
alpha.edge = 0.3)
```

```
netVisual_hierarchy2(cellchat@net$count,vertex.receiver =c(
  2:5
) ,
weight.scale = F,
vertex.weight = 0.5,
vertex.weight.max = 10,
vertex.size.max = 10,
edge.weight.max = 10,
edge.width.max = 0.1,
alpha.edge = 0.3)
mat <- cellchat@net$count
par(mfrow=c(2,4),xpd=T)
for (i in 1:nrow(mat)) {
  mat2    <-     matrix(0,nrow=nrow(mat),ncol=ncol(mat),dimnames     =
dimnames(mat))
  mat2[i,] <- mat[i,]

netVisual_circle(mat2,vertex.weight=groupSize,weight.scale=T,arrow.wid
th = 0.2,
                   arrow.size = 0.05,edge.weight.max = max(mat),
                   title.name = rownames(mat)[i],
  )
}
mat <- cellchat@net$weight
par(mfrow=c(2,4),xpd=T)
for (i in 1:nrow(mat)) {
  mat2    <-     matrix(0,nrow=nrow(mat),ncol=ncol(mat),dimnames     =
dimnames(mat))
  mat2[i,] <- mat[i,]

netVisual_circle(mat2,vertex.weight=groupSize,weight.scale=T,arrow.wid
th = 0.2,
                   arrow.size = 0.05,edge.weight.max = max(mat),
                   title.name = rownames(mat)[i],
  )
}
rankNet(cellchat,mode = 'single',stacked = T,do.stat = T)
cellchat@netP[["pathways"]]
pathways.show <- c("PERIOSTIN")
levels(cellchat@idents)
vertex.receiver=c( 1:5  )
netVisual_aggregate(cellchat,layout    =    'hierarchy',signaling    =
pathways.show,vertex.receiver = vertex.receiver)
par(mfrow=c(1,1))
```

```
netVisual_aggregate(cellchat,layout = 'circle',
                    signaling = pathways.show,
                    vertex.receiver = vertex.receiver,
                    edge.width.max = 30)
par(mfrow=c(1,1))
netVisual_aggregate(cellchat,layout = 'chord',signaling = pathways.show,vertex.receiver = vertex.receiver)
par(mfrow=c(1,1))
netVisual_heatmap(cellchat,signaling = pathways.show,color.heatmap = 'Reds')
netAnalysis_contribution(cellchat,signaling = pathways.show)
levels(cellchat@idents)

netVisual_bubble(cellchat,sources.use = c(1:5),
                 targets.use = c(6:8),
                 remove.isolate = F,font.size = 10)
RE_AP <- read.gmt('gmt/REACTOME_APOPTOSIS.v2023.2.Hs.gmt')
AP_genes <- c(RE_AP$gene)

AP_genes <- stringr::str_to_title(AP_genes)
AP_genes <- list(AP_genes[AP_genes%in%rownames(sce)])
sce.C <- AddModuleScore(sce.C,features = AP_genes,seed = 123,name = 'AP.Score')
colnames(sce.C@meta.data)[18] <- 'AP.Score'
FeatureDimPlot(
  srt = sce.C, features = c("AP.Score"),pt.size = 1,
  compare_features = F, label = F, label_insitu = F,
  reduction = "UMAP", theme_use = "theme_blank"
)
RidgePlot(sce.C, features = 'AP.Score',cols = mycol)
#load('1.2.RData')
```
