# Supplementary figures and images for: Single-cell transcriptomics in MI identify Slc25a4 as a new modulator of mitochondrial malfunction and apoptosis-associated cardiomyocyte subcluster
Source: Sci Rep. 2024 Apr 23;14:9274. doi: 10.1038/s41598-024-59975-8 (PMC11039722; doi:10.1038/s41598-024-59975-8)

Figure 8E

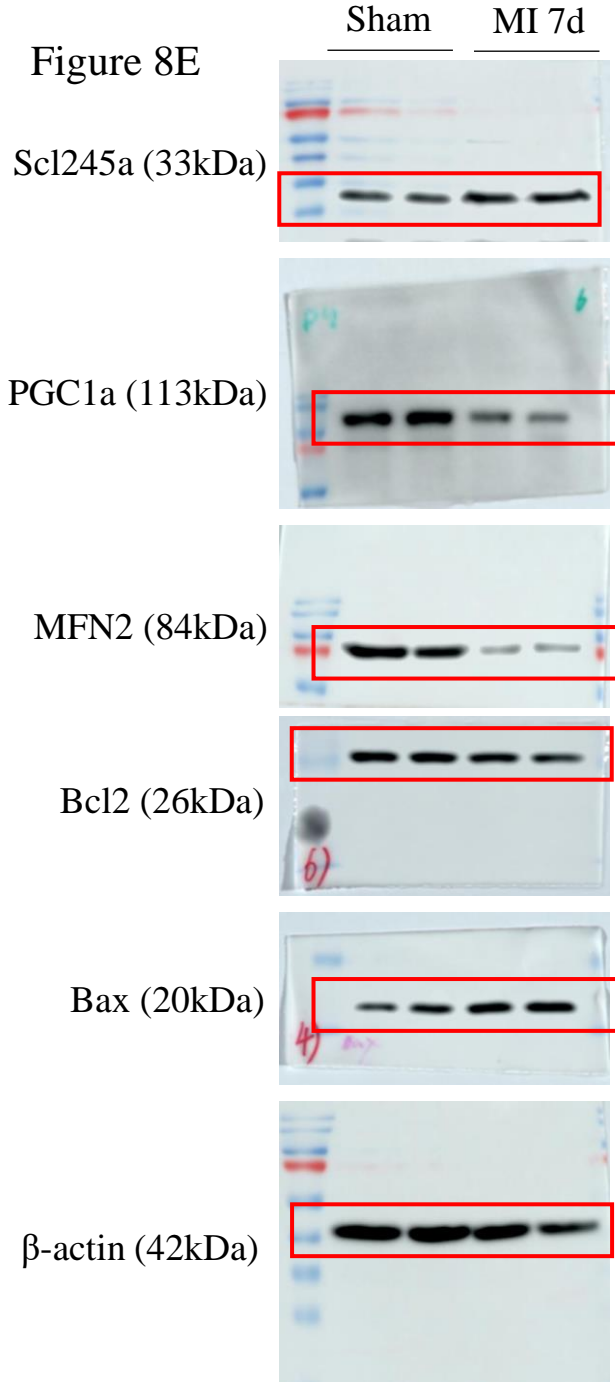

Figure 9A

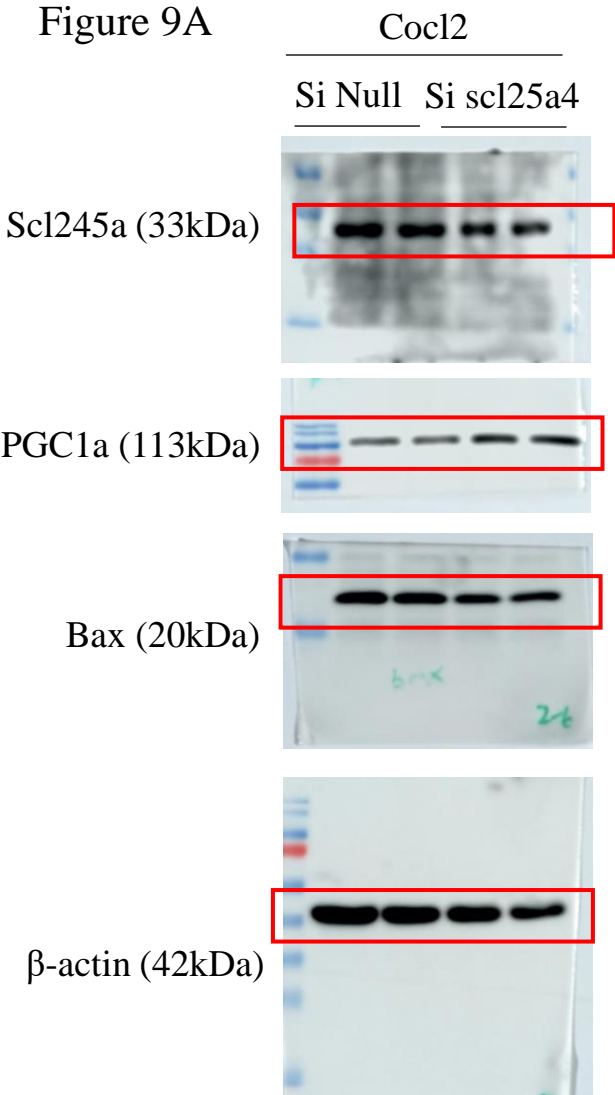

Figure 10C

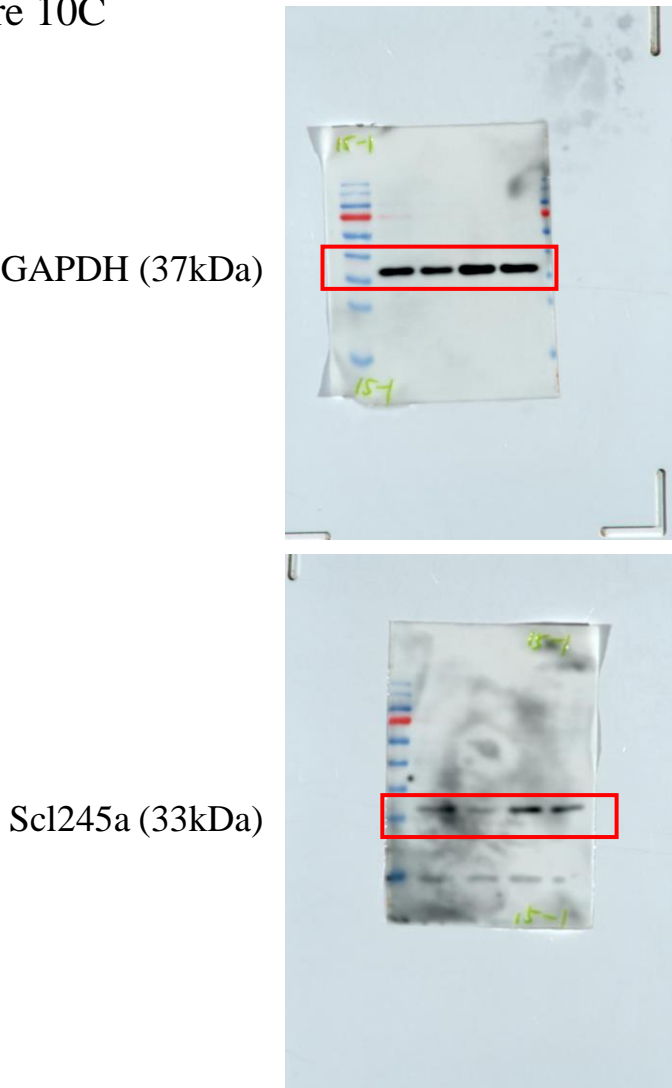

Figure 10C

GAPDH (37kDa)

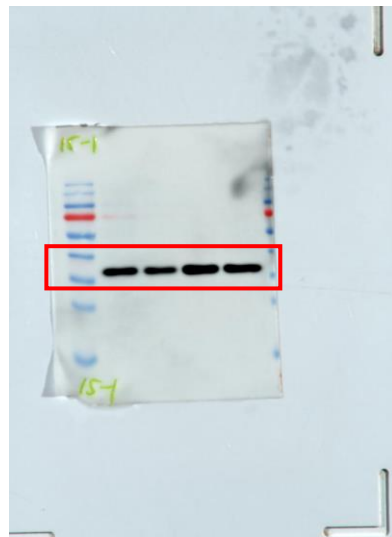

Scl245a (33kDa)

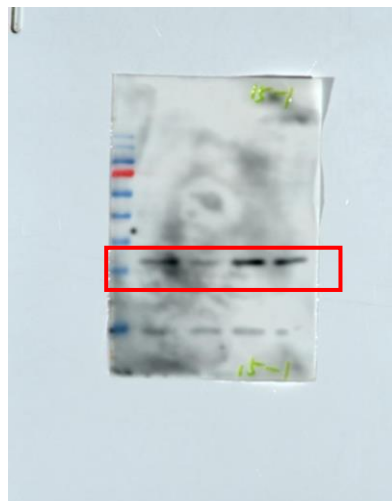

Supplement: Supplementary file 3 — Supplementary Figures. [file 41598_2024_59975_MOESM3_ESM.pdf]
